# Effectiveness of eHealth Interventions in Improving Medication Adherence Among Patients With Cardiovascular Disease: Systematic Review and Meta-Analysis

**DOI:** 10.2196/58013

**Published:** 2024-07-15

**Authors:** Yiqun Miao, Yuan Luo, Yuhan Zhao, Mingxuan Liu, Huiying Wang, Ying Wu

**Affiliations:** 1 School of Nursing Capital Medical University Beijing China

**Keywords:** eHealth, medication adherence, heart, cardiovascular, adherence, cardiology, digital health, cardiovascular diseases, network meta-analysis, synthesis, syntheses, review methods, review methodology, search, searches, searching, systematic, meta-analysis, meta-analyses, drug, drugs, pharmacy, pharmacies, pharmacology, pharmacotherapy, pharmaceutic, pharmaceutics, pharmaceuticals, pharmaceutical, medication, medications

## Abstract

**Background:**

Nonadherence to medication among patients with cardiovascular diseases undermines the desired therapeutic outcomes. eHealth interventions emerge as promising strategies to effectively tackle this issue.

**Objective:**

The aim of this study was to conduct a network meta-analysis (NMA) to compare and rank the efficacy of various eHealth interventions in improving medication adherence among patients with cardiovascular diseases (CVDs).

**Methods:**

A systematic search strategy was conducted in PubMed, Embase, Web of Science, Cochrane, China National Knowledge Infrastructure Library (CNKI), China Science and Technology Journal Database (Weipu), and WanFang databases to search for randomized controlled trials (RCTs) published from their inception on January 15, 2024. We carried out a frequentist NMA to compare the efficacy of various eHealth interventions. The quality of the literature was assessed using the risk of bias tool from the Cochrane Handbook (version 2.0), and extracted data were analyzed using Stata16.0 (StataCorp LLC) and RevMan5.4 software (Cochrane Collaboration). The certainty of evidence was evaluated using the Grading of Recommendations, Assessment, Development, and Evaluations (GRADE) approach.

**Results:**

A total of 21 RCTs involving 3904 patients were enrolled. The NMA revealed that combined interventions (standardized mean difference [SMD] 0.89, 95% CI 0.22-1.57), telephone support (SMD 0.68, 95% CI 0.02-1.33), telemonitoring interventions (SMD 0.70, 95% CI 0.02-1.39), and mobile phone app interventions (SMD 0.65, 95% CI 0.01-1.30) were statistically superior to usual care. However, SMS compared to usual care showed no statistical difference. Notably, the combined intervention, with a surface under the cumulative ranking curve of 79.3%, appeared to be the most effective option for patients with CVDs. Regarding systolic blood pressure and diastolic blood pressure outcomes, the combined intervention also had the highest probability of being the best intervention.

**Conclusions:**

The research indicates that the combined intervention (SMS text messaging and telephone support) has the greatest likelihood of being the most effective eHealth intervention to improve medication adherence in patients with CVDs, followed by telemonitoring, telephone support, and app interventions. The results of these network meta-analyses can provide crucial evidence-based support for health care providers to enhance patients’ medication adherence. Given the differences in the design and implementation of eHealth interventions, further large-scale, well-designed multicenter trials are needed.

**Trial Registration:**

INPLASY 2023120063; https://inplasy.com/inplasy-2023-12-0063/

## Introduction

Cardiovascular diseases (CVDs) encompass a range of disorders of the heart and blood vessels, including coronary heart disease, heart failure, aortic disease, cerebrovascular disease, and peripheral artery disease [1]. CVDs account for nearly one-third of global deaths, with approximately 17.9 million people dying annually [2]. Despite advancements in therapeutic modalities like angioplasty, pharmacological treatments remain the cornerstone for improving the prognosis and reducing the risk of major adverse cardiovascular events and mortality across all patient groups, regardless of whether they have undergone invasive interventions [3]. However, medication adherence among these patients is suboptimal, with about one-third not following prescribed regimens [4]. Poor medication adherence is concerning, as among self-reported nonadherent patients, the risk of mortality increases by 3.8 times [5]. In the United States, the preventable health care costs attributable to nonadherence exceed US $100 billion annually [6].

The challenges to medication adherence in patients with CVDs are multifaceted, primarily driven by forgetfulness, fear of side effects, communication gaps between patients and health care providers, socioeconomic factors, and lack of motivation [7]. In response to these challenges, eHealth interventions have gained attention as effective tools for enhancing preventive care across various settings [8]. eHealth encompasses diverse technological applications in health care, including digital information resources, remote monitoring, teleconsultations, and mobile-supported care [9]. These interventions facilitate real-time adjustments in health behavior to accommodate changes in individual health needs, interventional goals, and available resources [10]. Furthermore, eHealth technologies offer engaging, user-friendly, evidence-based solutions that can potentially reduce health care costs [11]. Previous studies have shown that eHealth technologies such as SMS text messaging, voice-enabled response systems, and phone calls have successfully provided feedback on medication adherence, significantly improving adherence rates among patients with chronic conditions [12,13]. Additionally, these interventions have proven effective in promoting lifestyle and behavioral changes necessary to manage blood pressure and cholesterol levels [14,15].

However, the concept of eHealth interventions is notably broad, encompassing a range of approaches that use various electronic devices. These interventions can vary greatly, from simple measures such as phone calls or emails to more comprehensive and integrated strategies that may include elements of automation and artificial intelligence [16]. Due to the diversity in the scope, components, and sophistication of eHealth interventions included in different studies, their effectiveness in enhancing medication adherence can also vary significantly [17]. Traditional meta-analyses or systematic reviews typically require that the interventions in the studies being compared are relatively consistent, which poses a challenge when attempting to compare the effects of different eHealth interventions [18,19]. Therefore, to address these variations and provide a more detailed understanding of how different eHealth interventions impact medication adherence among patients with CVDs, we conducted a network meta-analysis (NMA) on the existing evidence to rank the effects of various eHealth interventions on medication adherence among patients with CVDs, so as to provide evidence for clinical practice and health policy.

## Methods

### Protocol and Registration

The NMA adhered to the PRISMA-NMA (Preferred Reporting Items for Systematic Reviews and Meta-Analyses for Network Meta-Analyses) guidelines (Table S1 in Multimedia Appendix 1) [20]. It has been registered with the INPLASY, with the registration number INPLASY2023120063.

### Search Strategies

We screened several databases with no limitations on language or publication date, from inception to January 15, 2024. These databases included PubMed, Cochrane, Web of Science, Embase, China National Knowledge Infrastructure Library, Chinese Biomedical Literature Database, Chinese Scientific Journal database, and WanFang databases. To develop this comprehensive search strategy, we consulted with a librarian experienced in medical research and a cardiovascular expert to ensure a thorough search. The following Medical Subject Headings (MeSH) and keywords incorporating Boolean operators were applied: “cardiovascular disease,” “telemedicine,” “eHealth,” “smartphone,” “mobile applications,” “text messaging,” “medication adherence,” and “randomized controlled trials,” among others. Furthermore, manual searches in major international conference proceedings, systematic reviews, meta-analyses, and gray literature were carried out by 2 authors to systematically identify potential studies to prevent the omission of relevant studies, particularly those with only abstracts available. The search strategies are detailed in Textbox S1 in Multimedia Appendix 1.

### Inclusion and Exclusion Criteria

Inclusion criteria were based on the PICOS (Participants, Interventions, Comparison, Outcomes, and Study Design) criterion. Studies that satisfied the following criteria were included:

- Participants: patients were diagnosed with CVDs, including hypertension, ischemic heart disease, myocardial infarction, acute coronary syndrome, heart failure, and peripheral arterial disease.
- Interventions: trials had to include at least 1 type of eHealth intervention, such as telephone support, mobile phone apps, telemonitoring, SMS, or combined intervention (more than 2 types of eHealth interventions).
- Comparison: the comparative arms could include various interventions or usual care.
- Outcomes: the primary outcome was medication adherence, defined as the degree to which an individual’s medication-taking behavior aligns with the recommendations provided by health care providers [21]. Studies using the validated scale for evaluation were included. Secondary outcomes included systolic and diastolic blood pressure.
- Study design: The study design was restricted to RCTs.

We excluded studies that were single-arm studies, conference abstracts, letters to the editor, or study protocols that lacked adequate data for extraction.

After reviewing the interventions and discussion, we classified the interventions into the following categories:

- App intervention: Defined as an intervention delivered by an app on a mobile phone operating on iOS or Android OS.
- SMS text messaging intervention: Defined as any intervention using SMS text messaging to deliver content to the patient, with the capability for back-and-forth communication between fixed telephone and mobile telephone equipment.
- Telemonitoring intervention: Defined as the use of remote devices for long-distance monitoring of patients, with collected data transmitted to health care professionals. Guidance and interventions are provided through remote communication methods upon the detection of issues or the need for adjustments to the treatment plan.
- Telephone support intervention: Defined as coaching, medication reminders, or education delivered via telephone. This includes voice interaction mode and telephone follow-up.
- Combined intervention: Defined as incorporating more than 2 types of eHealth interventions.
- Usual care: Defined as the standard treatment and nursing provided by health care professionals, typically including the prescription of cardiovascular medications and lifestyle advice. This does not include the information reminders offered by eHealth technologies.

### Data Extraction

All initial search results were independently reviewed by 2 investigators. Duplicates and irrelevant articles were removed using EndnoteX9 (Thompson ISI Research Soft), based on titles and abstracts. Subsequently, full texts underwent comprehensive evaluation to ensure compliance with all inclusion and exclusion criteria. Any discrepancies were resolved through team discussions, with a third reviewer available for arbitration if necessary.

The data extracted included general information (authors, year of publication), participants’ characteristics (gender proportion, age range), study characteristics (duration of intervention, recruiting area, number of patients, type of intervention), and methods of measuring medication adherence. We reached out to the authors for any missing data. Our primary focus was on medication adherence resulting from adopting eHealth interventions, using various validated scales (Table S2 in Multimedia Appendix 1 [22-26]). We extracted mean differences and SDs between baseline and the last observation in order to calculate the change score for medication adherence.

### Risk of Bias and Certainty of Evidence Assessment

The methodological quality of each study using the Cochrane Risk of Bias tool 2.0 [27], categorizing studies into low, high, or unclear risk of bias, was independently assessed by 2 reviewers. The assessment covered several aspects, including random sequence generation, allocation concealment, blinding of participants and personnel, incomplete outcome data, selective reporting, and other biases. In evaluating the outcomes of the NMA, we used the GRADE framework to assess the evidence level [28]. This framework classifies the risk of bias, inconsistency, indirectness, imprecision, and publication bias for each paired comparison into 4 categories: “high,” “moderate,” “low,” or “very low.” Discrepancies were resolved by a third-party investigator.

### Statistical Analysis

The NMA was performed using Stata (version 16.0; StataCorp LLC). A network plot visually represented the primary evidence for each eHealth intervention. Each node represented an eHealth intervention, with its size dependent on the number of patients directly involved. These nodes are connected by lines of varying thickness, which indicate whether there is a direct relationship between the interventions. Additionally, the thickness of the lines is weighted based on the direct evidence between them. We used standardized mean differences (SMDs) and 95% CIs to pool data from various adherence scales to ensure adequate comparability. The mean differences and SD were either directly extracted from the published data or computed using the available information [29]. Studies were evaluated for heterogeneity using the *I*^2^ statistic, with low, medium, and high degrees of heterogeneity indicated by values of approximately 25%, 50%, and 75%, respectively [30]. Subgroup analyses were conducted to explore the effect of gender (males vs females), duration of intervention (≥3 months vs <3 months), and mode of delivery (theory-driven intervention vs interventions not based on theory).

It is crucial to consider network transitivity in NMA, as it serves as a foundational assumption that significantly impacts our subsequent analysis [31]. To ensure the validity of indirect inferences among the various treatment comparisons, we assessed the transitivity assumption by examining clinical and methodological characteristics, including patient profiles and study designs, across all studies included in our analysis. Surface under the cumulative ranking (SUCRA) probabilities were calculated to rank each intervention’s efficacy compared to an ideal intervention that always performs the best without uncertainty. SUCRA scores range from 0 to 1, with a higher score indicating a higher likelihood that an intervention is the most effective [32]. We conducted node-splitting analysis to assess the inconsistency between direct and indirect evidence estimates for each intervention comparison [33]; in cases without significant inconsistency (*P*>.05), a consistency model was applied. A comparison-adjusted funnel plot was created to identify potential sources of bias.

## Results

### Participants and Study Characteristics

**Figure 1:**
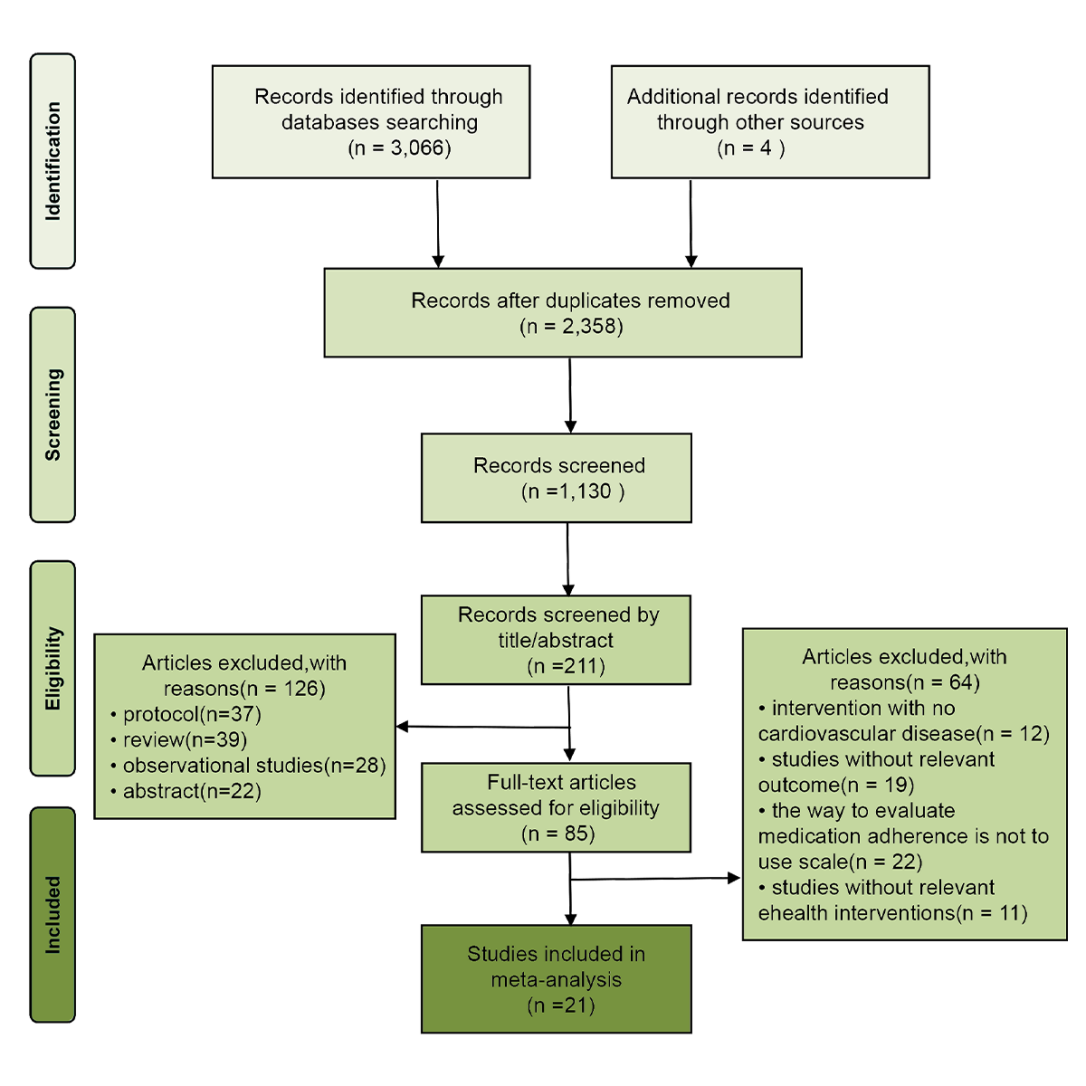
Flowchart of study selection process.

The literature screening flowchart for our NMA is depicted in Figure 1. Initially, 3066 publications were identified through database searches, and 4 additional studies were found via manual searches. Of the 85 full-text articles evaluated for eligibility, 21 studies [34-54] were ultimately included. These studies involved a total of 3804 participants, divided into intervention (n=1937) and control (n=1867) groups. The intervention types were as follows: 4 studies used app interventions [38-41], 4 used telephone support [42-44], 4 used telemonitoring interventions [34-37], 6 used SMS text messaging interventions [45-50], and 4 used combined interventions [51-54]. Participants ranged in age from 35 to 80 years, with 51.3% being female. The duration of the interventions varied from 1 to 8 months, and the studies were conducted across various regions, including 7 studies in North America, 9 in Asia, and 5 in Europe and other areas. The characteristics of these studies are detailed in Table 1.

The risk of bias assessment is depicted in Figure S1 in Multimedia Appendix 1. Regarding random sequence generation, 20 RCTs exhibited low bias, and 19 RCTs showed low bias in allocation concealment. Performance bias was a concern in 1 RCT assessed as high risk and in 7 RCTs assessed with unclear risk levels. Detection bias was observed in 5 RCTs with high overall risks and in 6 with unclear risks. The GRADE assessment for outcomes is shown in Tables S3-S5 in Multimedia Appendix 1. Certainty-in-effect estimates for each comparison were displayed, and the reasons for downgrading were also shown.

**Table 1:** Characteristics of included studies (N=3804).

Reference	Participants (n), intervention/control	Age (years), mean (SD)		Proportion of female participants (%)	Recruiting area	Study population	Intervention or control	Duration of intervention	Scale of medication adherence
		Intervention	Control						
									
He et al 2022 [38]	41/41	NR^a^	NR	51.2	China	Cardiovascular disease	App vs usual care	8 weeks	SEAMS^b^
Zha et al 2020 [34]	12/13	48.90 (8.00)	55.50 (5.20)	88.0	USA	Hypertension	Telemonitoring vs usual care	6 months	MASES^c^
Kim et al 2016 [35]	52/43	57.50 (8.60)	57.70 (8.70)	68.4	USA	Hypertension, cardiac arrhythmia	Telemonitoring vs usual care	6 months	MMAS-8^d^
Yanicelli et al 2020 [39]	15/15	NR	NR	20.0	Argentina	Heart failure	App vs usual care	90 days	MMAS-8
Sarfo et al 2018 [36]	30/30	54.30 (11.90)	55.90 (13.70)	35.0	Ghana	Stroke survivors	Telemonitoring vs SMS	3 months	MMAS-8
Chandler et al 2019 [37]	26/28	44.40 (7.20)	46.80 (8.10)	64.8	USA	Essential hypertension	Telemonitoring vs usual care	2 months	MMAS-8
Morawski et al 2018 [40]	209/202	51.70 (10.50)	52.40 (10.10)	60.1	USA	Hypertension	App vs usual care	12 weeks	MMAS-8
Santo et al, 2019 [41]	107/56	58.40 (9.04)	56.80 (8.64)	12.5	Australia	Coronary heart disease	App vs usual care	3 months	MMAS-8
Wan et al 2016 [42]	40/40	59.07 (12.36)	60.24 (12.57)	28.8	China	Ischemic stroke patients	Telephone support vs usual care	6 months	MMAS-4^e^
Migneault et al 2012 [43]	169/168	59.17 (12.63)	58.44 (12.59)	70.3	USA	Hypertension	Telephone support vs usual care	8 months	MMAS-7^f^
Najafi et al 2016 [44]	50/50	58.92 (9.64)	57.70 (10.64)	54.0	Iran	Myocardial infarction	Telephone support vs usual care	12 weeks	MMAS-8
Buis et al 2017 [45]	65/58	46.30 (8.00)	52.20 (7.60)	55.4	USA	Hypertension	SMS vs usual care	1 month	MMAS-8
Bermon et al 2021 [46]	462/468	64.00 (9.70)	63.10 (10.00)	21.6	Colombia	Cardiovascular disease	SMS vs usual care	52 weeks	MARS-5^g^
Bhandari et al 2022, [47]	100/100	49.20 (9.78)	51.70 (9.21)	44.5	Nepal	Hypertension	SMS vs usual care	12 weeks	MASES
Park et al 2014 [48]	28/28	58.20 (10.60)	61.60 (9.10)	30.4	USA	Coronary heart disease	SMS vs usual care	30 days	SEAMS
Kamal et al 2015 [49]	100/100	56.07 (1.50)	57.62 (1.30)	32.5	Pakistan	Stroke	SMS vs usual care	2 months	MMAS-8
Zhai et al 2020 [50]	192/192	68.50 (7.90)	69.40 (9.70)	69.0	China	Hypertension	SMS vs usual care	3 months	MMAS-8
Wan et al 2018 [51]	80/78	35.00-86.00^h^	35.00-86.00^h^	34.8	China	Hypertensive ischemic stroke	Combined intervention^i^ vs telephone support	3 months	HPLP II^j^
Kes and Polat 2022 [52]	39/38	54.90 (6.60)	52.20 (6.20)	53.2	Turkey	Hypertension	Combined intervention^k^ vs usual care	3 months	MASES
Kamal et al 2018 [53]	99/98	59.10 (11.60)	57.70 (11.10)	22.8	Pakistan	Stroke and heart attack survivors	Combined intervention^l^ vs usual care	3 months	MMAS-8
Schoenthaler et al 2020 [54]	21/21	59.70 (10.70)	57.60 (11.10)	45.2	USA	Uncontrolled hypertension	Combined intervention^m^ vs usual care	3 months	MMAS-8

^a^NR: not reported.

^b^SEAMS: Self-efficacy for appropriate medication use scale.

^c^MASES: Medication adherence self-efficacy scale.

^d^MMAS-8: Morisky Medication Adherence Scale–8.

^e^MMAS-4: Morisky Medication Adherence Scale–4.

^f^MMAS-7: Morisky Medication Adherence Scale–7.

^g^MARS-5: Medication Adherence Report Scale–5.

^h^Range.

^i^Combined intervention: SMS and telephone support.

^j^HPLP II: Health Promoting Lifestyle Profile II.

^k^Combined intervention: SMS and telephone support.

^l^Combined intervention: SMS and telephone support.

^m^Combined intervention: Tablet-based intervention, text message interactions, and video education.

### Primary Outcome

The pairwise meta-analyses demonstrated that the efficacy of the combined intervention was superior to that of telephone support, app interventions, and telemonitoring interventions (Figure S2 in Multimedia Appendix 1). *I*^2^ values reflected that our preliminary meta-analysis revealed a high degree of heterogeneity among all the included studies (*I*^2^=78.4%, *P*<.001).

**Figure 2:**
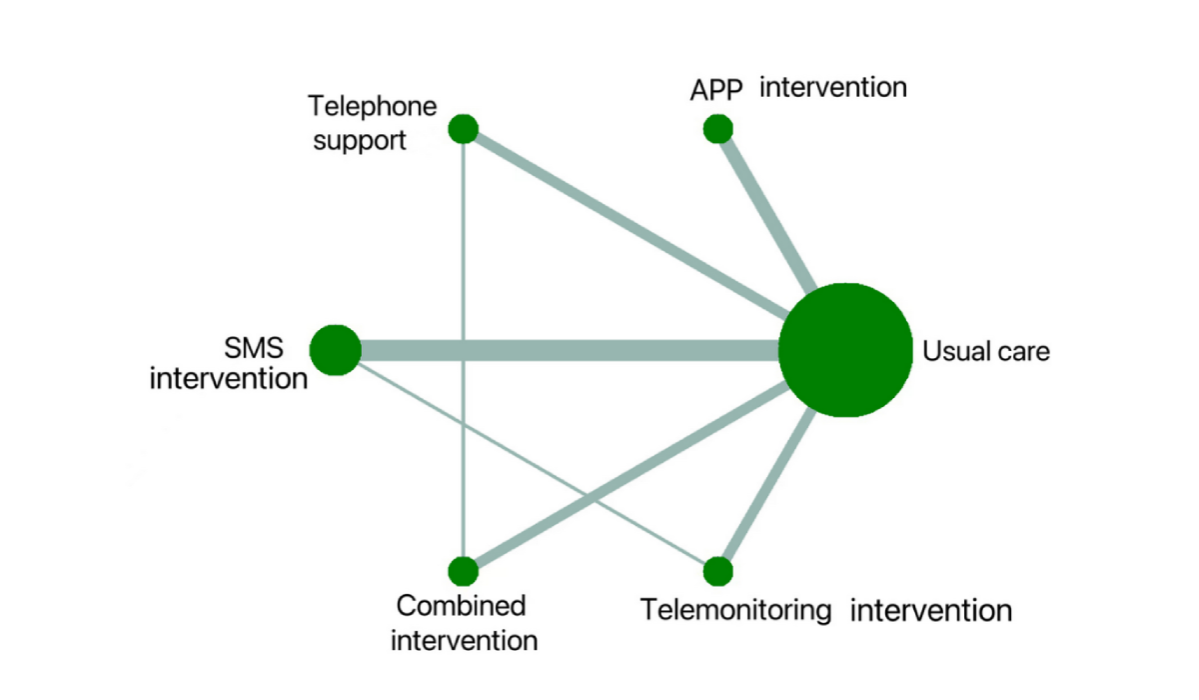
The network plot regarding medication adherence. App, mobile phone applications; SMS, short messaging service.

**Figure 3:**
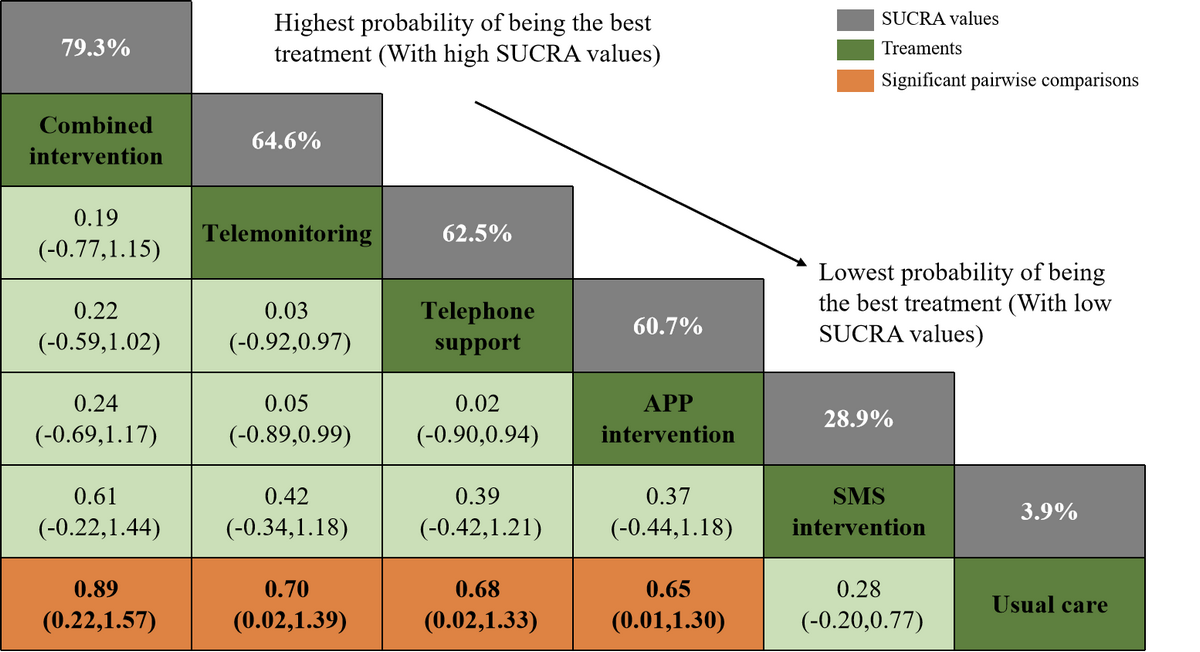
Relative effect sizes of medication adherence at postintervention according to network meta-analysis. App, mobile phone applications; SMS, short messaging service.

Figure 2 illustrates the network evidence for medication adherence outcomes. The analysis included 7 direct and 8 indirect comparisons among the eHealth interventions. The size of the nodes indicated that usual care involved the most participants (n=1759), while the combined intervention had the fewest (n=120). Figure 3 depicts the relative effect sizes of medication adherence post intervention. The following 4 eHealth interventions were statistically superior to usual care in enhancing medication adherence: the combined intervention (SMD 0.89, 95% CI 0.22-1.57), telemonitoring intervention (SMD 0.70, 95% CI 0.02-1.39), telephone support intervention (SMD 0.68, 95% CI 0.02-1.33), and app intervention (SMD 0.65, 95% CI 0.01-1.30). However, the SMS text messaging intervention did not show a significant difference from usual care (SMD 0.28, 95% CI –0.20 to 0.77; low to very low certainty). The hierarchy of each eHealth intervention was ranked according to SUCRA values. The combined intervention (SUCRA 79.3%) possessed the greatest likelihood of being the best intervention for medication adherence, along with the suboptimal telemonitoring intervention (SUCRA 64.6%), followed by telephone support (SUCRA 62.5%), app (SUCRA 60.7%), and SMS text messaging interventions (SUCRA 28.9%); usual care had the lowest SUCRA (3.9%), as shown in Figure S3 in Multimedia Appendix 1.

**Figure 4:**
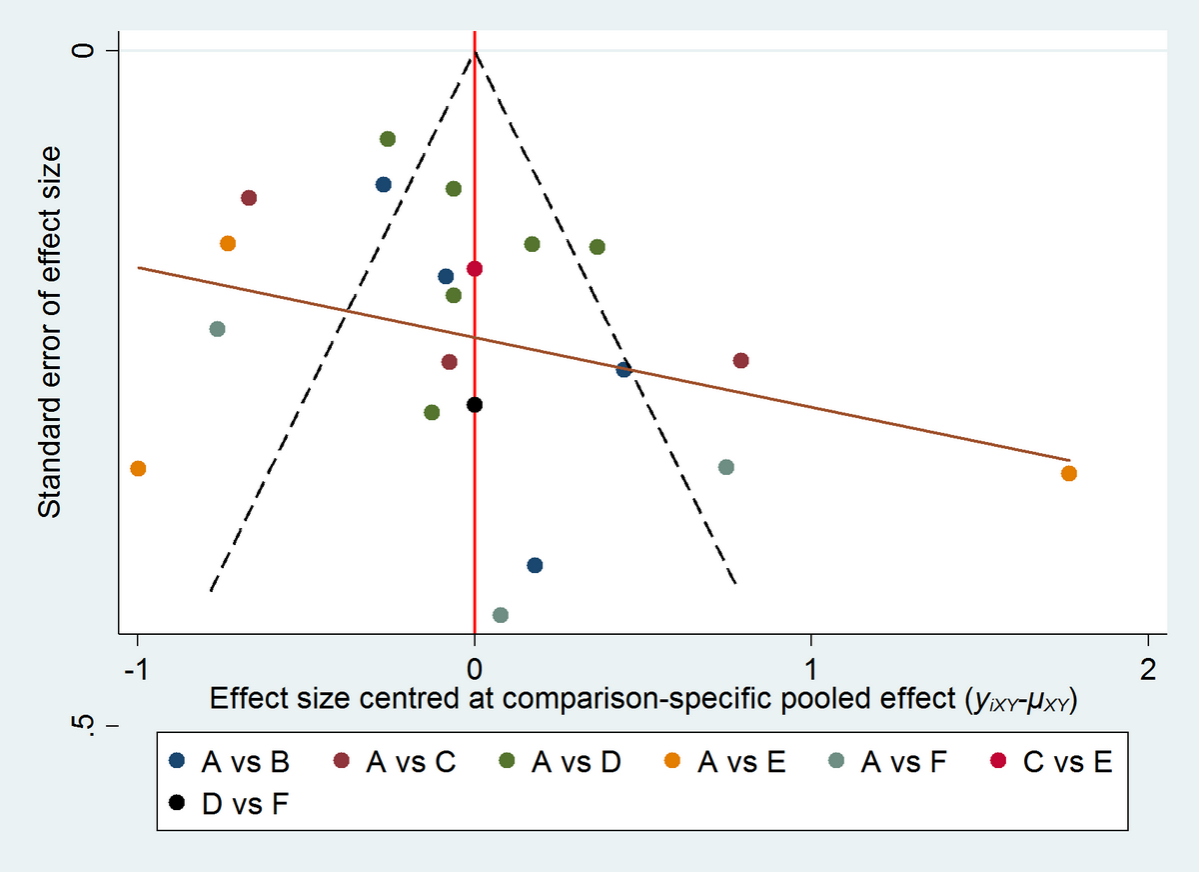
Funnel plot of medication adherence. (A) Usual care. (B) Mobile phone applications intervention. (C) Telephone support intervention. (D) Short messaging service intervention. (E) Combined intervention. (F) Telemonitoring intervention.

The node-splitting method revealed no local inconsistency between direct and indirect evidence among the eHealth interventions (*P*≥.05). The loop inconsistency plot formed a closed loop among different interventions, as shown in Figure S4 in Multimedia Appendix 1. Figure 4 displayed an inverted funnel plot with several scattered points asymmetrically distributed, suggesting a certain level of publication bias.

### Secondary Outcomes

In terms of systolic blood pressure (SBP), 11 studies with 2608 participants were included in the analysis. The combined interventions emerged as the only method significantly more effective than usual care for lowering SBP. Specifically, it surpassed SMS text messaging interventions (SMD –1.08, 95% CI –2.15 to –0.01) and usual care (SMD –1.21, 95% CI –2.12 to –0.31). There was no statistical difference in app, telephone support, telemonitoring, and SMS text messaging interventions compared with the usual care group. The ranking of interventions based on their efficacy revealed that the combined interventions (89.4%) had the highest probability of being the most effective, followed by the app (67.0%), telephone support (61.1%), telemonitoring (35.3%), and SMS text messaging (30.0%) interventions, and usual care (17.2%; moderate to low certainty).

Regarding diastolic blood pressure (DBP), 10 studies with a total of 2197 participants were analyzed. The combined interventions were the only ones to outperform usual care statistically. It was more effective than SMS text messaging interventions (SMD 0.90, 95% CI –1.70 to –0.10), usual care (SMD –0.95, 95% CI –1.64 to –0.27), and telemonitoring interventions (SMD –1.13, 95% CI –2.11 to –0.16) in reducing DBP. There was no statistical difference in app, telephone support, telemonitoring, and SMS text messaging interventions compared with the usual care group. The results of intervention ranking showed that combined interventions (92.9%) had the highest probability of being the best intervention, followed by telephone support (70.9%), app (49.3%), SMS text messaging (37.2%), usual care (30.2%), and telemonitoring (19.2%; moderate to low certainty). The network plots for SBP and DBP were provided in Figure S5 in Multimedia Appendix 1. Table S6 in Multimedia Appendix 1 detailed the relative effect sizes of SBP and DBP at postintervention according to the NMA, and the SUCRA rankings were presented in Figure S6 in Multimedia Appendix 1. The node-splitting method found no significant inconsistencies between direct and indirect evidence among the eHealth interventions (*P*≥.05). The funnel plot of SBP and DBP, shown in Figure S7A and B in Multimedia Appendix 1, indicated no evidence of publication bias.

### Subgroup Analysis

In subgroups of patients with interventions lasting ≥3 months, the efficacy levels were similar to the main analysis, with the combined intervention significantly outperforming others for improving medication adherence. In contrast, for interventions lasting <3 months, the telephone support intervention emerged as potentially the most effective. The combined interventions demonstrated a significant benefit in medication adherence for males (SMD 2.60, 95% CI 1.06-4.13), while the app interventions showed significant benefits for females (SMD 0.76, 95% CI 0.29-1.23) compared to usual care. Subgroup analysis revealed that among theory-driven eHealth interventions, the telemonitoring intervention is the most effective. For interventions not based on theory, the combined intervention was the most effective. The relative effect sizes of these subgroup analyses for duration of intervention, gender, and theory-based interventions post intervention according to the NMA are detailed in Table S7 A, B, and C in Multimedia Appendix 1.

## Discussion

### Principal Findings

To the best of our knowledge, this is the first meta-analysis to assess the comparative effectiveness of eHealth in improving medication adherence among patients with CVDs. The outcomes from direct and indirect comparisons were combined, and various eHealth interventions were quantitatively ranked to identify the optimal protocol. This process laid the foundation for choosing the appropriate eHealth interventions to enhance medication adherence. This systematic review determined that combined interventions, telephone support, telemonitoring interventions, and app interventions appear to be effective in improving medication adherence in individuals with CVDs. Notably, the combined interventions had a 79.3% probability of being the most effective for improving medication adherence. Additionally, combined interventions provided the greatest benefit for the outcome of SBP and DBP. The research outcomes underscore the importance of incorporating multimodal functionalities in the development of eHealth interventions.

In our study, the combined intervention proved to be the most effective method for enhancing medication adherence. This aligns with Ding et al’s [55] findings, which highlight that medication adherence in patients is influenced by multiple factors, necessitating a combination of various approaches for a comprehensive intervention strategy. The effectiveness of combined interventions can be attributed to their tailored nature, offering personalized measures attuned to different disease stages. For example, employing telephone interventions for timely feedback during early stages and using automated SMS text messaging interventions for long-term reminders in later stages [51-53]. Additionally, the study of Schoenthaler et al [54] used tablet-based interventions to identify patients’ most prominent adherence barriers, employing SMS text message interactions and video education to help alleviate these barriers. The findings reinforce the notion that in the design of electronic interventions, it is advisable to use multimodal functionalities. Such an approach enables the delivery of tailored, comprehensive treatment plans that are best suited to the unique requirements of each patient. Additionally, several studies [56,57] have explored the integration of smart pillboxes with telephones or apps for tracking whether the pillbox has been opened and providing reminders to patients, aiming to mitigate adherence issues associated with forgetfulness. However, these studies use internal algorithms to calculate adherence rates that are not standardized, making it difficult to combine and compare adherence data from different studies in our meta-analysis. Meanwhile, technological advancements have facilitated the development of complex customized algorithms. Ecological momentary assessments allow for real-time monitoring of patients’ behaviors and experiences [58]. User personas provide a detailed understanding of patients’ characteristics, preferences, and needs [59]. Studies have indicated that integrating these methods as auxiliary support in eHealth is a significant method for providing precise nursing to patients. However, most studies on these technologies focus on technology development and user acceptance, lacking RCTs to validate their efficacy, and were not included in our meta-analysis, highlighting them as potential areas for future research.

This study also highlights the differential effectiveness of various eHealth modalities. Telemonitoring interventions, probably due to their features like audiovisual and alarm support for medication adherence, appear superior to telephone support and app interventions, although telephone support and app interventions were also effective compared to usual care in this analysis. The underlying mechanisms are unclear, but multifaceted functions of the modality might contribute to its effectiveness in improving medication adherence. Audiovisual features can help patients better understand their condition and treatment plans, thereby enhancing their engagement and willingness to follow medical advice. With an alarm function, it can alert patients when medication nonadherence is detected, reducing the instances of forgetting or incorrectly taking medication and assisting in the formation of a habit of taking medication on schedule. Moreover, telemonitoring interventions focus on real-time biometric data tracking and remote clinical intervention, which facilitates rapid access to professional guidance for patients when needed [60]. In contrast, app interventions typically provide textual medication reminders and are more oriented toward encouraging self-management among patients with CVDs, thereby increasing the treatment burden on patients [12]. In addition, our research targets middle-aged and older individuals, with an average age of 56.47, who possess lower health information literacy and find it challenging to keep pace with technological advancements [61]. Coupled with the complex interfaces and small font sizes in app interventions, this renders it challenging for them to effectively engage with these apps [62]. Therefore, designers of eHealth services should aim to develop more user-friendly interfaces and establish clearer information retrieval models by incorporating features such as larger fonts, voice recognition functionality, and simplified navigation. Despite those disadvantages, telephone support and app interventions could also improve medication adherence when compared with usual care.

It should be noted that SMS text messaging interventions alone were not supported by our NMA as an effective tool in improving medication adherence. This limitation may be due to SMS text messaging interventions being a unidirectional form of communication. Among the 5 articles we included that focused on SMS text messaging interventions, only 1 study required participants to respond to SMS text messages within 6 hours to confirm whether they had taken their medication, while the others used automated, scheduled messages. As Yasmin et al [63] point out, communication between health care providers and patients can alter patients’ attitudes and behaviors toward disease management. This reminds us that providers not only need to offer informational support but also emotional support, thereby enhancing the patient’s sense of participation and experience. The recommendation for a 2-way SMS text messaging system aligns with a global directive, yet the discreet nature of SMS text messaging should not be overlooked as it offers confidentiality and reduces stigma surrounding the receipt of treatment among patients [64,65]. It should also be noted that the influence of eHealth on patient medication adherence is not instantaneous but is gradually formed through improving their health information efficacy and health awareness [16].

This review shows that objective measures of blood pressure can be improved with combined eHealth interventions. A plausible reason may be that the enhancement of medication adherence via eHealth and the reduction of blood pressure are mutually reinforcing. A single explanation cannot fully capture the influence of combined eHealth interventions on blood pressure, as it is probable that they result from a combination of psychological aspects (such as self-efficacy, encouragement mechanisms) and physiological mechanisms. eHealth encourages patients to adopt regular medication routines and other positive lifestyle modifications, thereby enhancing the efficacy of blood pressure control and achieving the expected treatment outcomes. A previous review [66] also similarly indicated that eHealth in conjunction with other modes significantly reduced blood pressure. In addition, eHealth facilitates a transition from passive treatment to active prevention by continuously monitoring users’ health status. This prevention-centric health care model contributes to reducing the frequency of outpatient visits, thereby saving on health care costs [18]. However, among the screened trials, there were no studies assessing the economic benefits of using eHealth interventions to improve medication adherence. This highlights the need for further research to understand their role in improving health outcomes while saving costs.

In the subgroup analysis, the combined interventions exhibited stronger efficacy in patients with an intervention time ≥3 months, which was similar to the results in the main analysis. Conversely, telephone support showed greater efficacy for intervention periods <3 months. A possible explanation is that combined interventions may provide patients with more comprehensive support, including informational education, reminder services, and behavioral incentives, which are particularly effective in long-term interventions. In contrast, for short-term interventions, the directness and convenience of telephone support may be more acceptable to patients [67].

It is worth noting that approximately 48% of the studies in this review reported the use of theory-driven design, such as self-determination theory and cognitive behavioral theory, to guide their eHealth interventions. In the subgroup of theory-driven eHealth interventions, telemonitoring intervention ranked first. Combined intervention became the most effective for patients among all interventions that are not based on theory. When interventions are theory-driven, telemonitoring interventions may be tailored more precisely and personalized, focusing on the behaviors and motivations of target users. In contrast, a diversified approach might be more adaptable to the needs and preferences of different patients, especially in situations lacking theory-driven guidance. Research indicates that employing theory-driven approaches to developing eHealth interventions may not only enhance our arsenal of effective strategies but also potentially enrich our theoretical and practical insights into changing health behaviors [68]. Therefore, to develop optimal eHealth interventions, it is essential to establish specific behavioral objectives and hypothesized mechanisms of action [69].

### Limitations

Among the limitations of this study is the fact that, despite our careful consideration in classifying eHealth interventions, the classifications may not be entirely accurate due to the diversity of components and settings inherent in eHealth devices. Furthermore, we were unable to perform subgroup analyses based on specific types of CVDs many studies did not focus exclusively on a single condition. This limited our ability to conduct a more in-depth analysis of differences in medication adherence among patients with different CVDs. Moreover, our research did not account for differences in medication adherence among the various drugs included in the studies. Factors such as side effects and the palatability of medications could contribute to varying rates of nonadherence. Finally, given the lack of a gold standard for assessing medication adherence, our study excluded those that did not use validated scales to measure adherence. This exclusion may have led to the omission of some relevant studies, potentially affecting the comprehensiveness of our analysis.

### Conclusions

To summarize, the research identifies combined interventions (SMS text messaging and telephone support) as the most effective eHealth method for improving medication adherence in patients with CVDs, followed in effectiveness by telemonitoring, telephone support, and app interventions. These findings could guide future practice-based interventions aimed at optimizing patient medication adherence. However, it is important to note that existing intervention protocols differ in terms of timing and specific strategies, necessitating further research to demonstrate the effectiveness of different eHealth interventions.

## Appendix

Multimedia Appendix 1 Supplementary tables and figures.

## Abbreviations

CNKI: China National Knowledge Infrastructure Library
CVD: cardiovascular disease
DBP: diastolic blood pressure
GRADE: Grading of Recommendations, Assessment, Development, and Evaluations
PICOS: Participants, Interventions, Comparison, Outcomes, and Study Design
PRISMA-NMA: Preferred Reporting Items for Systematic Reviews and Meta-Analyses for Network Meta-Analyses
RCT: randomized controlled trial
SBP: systolic blood pressure
SMD: standardized mean difference
SUCRA: surface under the cumulative ranking

## Data Availability

All data generated or analyzed during this study are included in the supplementary files accompanying this published article.
