# Supplementary material for: Effectiveness of eHealth Interventions in Improving Medication Adherence Among Patients With Cardiovascular Disease: Systematic Review and Meta-Analysis
Source: J Med Internet Res. 2024 Jul 15;26:e58013. doi: 10.2196/58013 (PMC11287104; doi:10.2196/58013)
Supplement: Multimedia Appendix 1 [file jmir_v26i1e58013_app1.docx]

**Multimedia Appendix 1. Comprehensive Collection of Tables and Figures**

**Textbox S1. Search strategies.**

Pubmed 516

#1 (Cardiovascular Disease[Mesh])) OR (Disease, Cardiovascular[Title/Abstract]) OR (Cardiac Events[Title/Abstract])) OR (Adverse Cardiac Event[Title/Abstract]))) OR (Coronary Heart Disease[Mesh]) OR (Coronary Disease[Title/Abstract])) OR (Angina Pectoris[Title/Abstract])) OR (atrial fibrillation[Title/Abstract])) OR (myocardial infarction[Title/Abstract])) OR (myocardial ischemia[Title/Abstract])) OR (heart failure[Title/Abstract])) OR (stroke[Title/Abstract])) OR (Cerebrovascular Disorders[Title/Abstract])) OR (peripheral arterial disease[Title/Abstract])) OR (peripheral vascular disease[Title/Abstract])) 2908359

#2 (telemedicine[Mesh]) OR (Telemonitoring[Title/Abstract])) OR (telephone[Title/Abstract])) OR (eHealth[Title/Abstract])) OR (Telehealth[Title/Abstract])) OR (mHealth[Title/Abstract])) OR (Mobile Health[Title/Abstract])) OR (Virtual Medicine[Title/Abstract])) OR (Medicine, Virtual[Title/Abstract])) OR (smartphone[Mesh])) OR (smart phone[Title/Abstract]) OR (Phones, Smart[Title/Abstract])) OR (Mobile Applications[Mesh])) OR (Mobile Apps[Title/Abstract])) OR (Portable Software Apps[Title/Abstract])) OR (Text Messaging[Mesh]) OR (text messages[Title/Abstract])) OR (Texting[Title/Abstract]))) 150960

#3 (randomized controlled trial[pt] OR controlled clinical trial[pt] OR randomized[tiab] OR placebo[tiab] OR clinical trials as topic[mesh:noexp] OR randomly[tiab] OR trial[ti]) NOT (animals [mh] NOT (humans [mh] AND animals[mh])) 739840

#4 (Medication Adherence[Mesh]) OR (Medication Compliance[Title/Abstract]) 27384

#5 #1 AND #2 AND #3 516

Embase 535

#1 'cardiovascular disease'/exp OR 'disease, cardiovascular':ti,ab OR 'cardiac events':ti,ab OR 'adverse cardiac event':ti,ab OR 'coronary heart disease'/exp OR 'coronary disease':ti,ab OR 'angina pectoris':ti,ab OR 'atrial fibrillation':ti,ab OR 'myocardial infarction':ti,ab OR 'myocardial ischemia':ti,ab OR 'heart failure':ti,ab OR 'stroke':ti,ab OR 'cerebrovascular disorders':ti,ab OR 'peripheral arterial disease':ti,ab OR 'peripheral vascular disease':ti,ab 5554136

#2 'telemedicine'/exp OR 'telemonitoring':ti,ab OR 'telephone':ti,ab OR 'ehealth':ti,ab OR 'telehealth':ti,ab OR 'mhealth':ti,ab OR 'mobile health':ti,ab OR 'virtual medicine':ti,ab OR 'medicine, virtual':ti,ab OR 'smartphone'/exp OR 'smart phone':ti,ab OR 'phones, smart':ti,ab OR 'mobile applications'/exp OR 'mobile apps':ti,ab OR 'portable software apps':ti,ab OR 'Text Messaging'/exp OR 'text messages':ti,ab OR 'texting':ti,ab 216432

#3 'crossover procedure':de OR 'double-blind procedure':de OR 'randomized controlled trial':de OR 'single-blind procedure':de OR (random* OR factorial* OR crossover* OR cross NEXT/1 over* OR placebo* OR doubl* NEAR/1 blind* OR singl* NEAR/1 blind* OR assign* OR allocat* OR volunteer*):de,ab,ti 3165172

#4 'medication Adherence'/exp OR 'medication Compliance':ti,ab 48456

#5 #1 AND #2 AND #3 AND #4 535

Cochance 900

#1 MeSH descriptor: (Cardiovascular Disease) OR (Disease, Cardiovascular):ti,ab,kw OR (Cardiac Events):ti,ab,kw OR (Adverse Cardiac Event):ti,ab,kw OR MeSH descriptor: (Coronary Heart Disease) OR (Coronary Disease):ti,ab,kw OR (Angina Pectoris):ti,ab,kw OR (atrial fibrillation):ti,ab,kw OR (myocardial infarction):ti,ab,kw OR (myocardial ischemia):ti,ab,kw OR (heart failure):ti,ab,kw OR (stroke):ti,ab,kw OR (Cerebrovascular Disorders):ti,ab,kw OR (peripheral arterial disease):ti,ab,kw OR (peripheral vascular disease):ti,ab,kw 200162

#2 MeSH descriptor: (telemedicine) OR (Telemonitoring):ti,ab,kw OR (telephone):ti,ab,kw OR (eHealth):ti,ab,kw OR (Telehealth):ti,ab,kw OR (mHealth):ti,ab,kw OR (Mobile Health):ti,ab,kw OR (Virtual Medicine):ti,ab,kw OR (Medicine, Virtual):ti,ab,kw OR MeSH descriptor: (smartphone) OR (smart phone):ti,ab,kw OR (Phones, Smart):ti,ab,kw OR MeSH descriptor: (Mobile Applications) OR (Mobile Apps):ti,ab,kw OR (Portable Software Apps):ti,ab,kw OR MeSH descriptor: (Text Messaging) OR (text messages):ti,ab,kw OR (Texting):ti,ab,kw 488154

#3(medication Adherence):ti,ab,kw OR (medication compliance):ti,ab,kw 33040

#4 #1 AND #2 900

Web of science 964

#1 TS=(cardiovascular disease OR disease, cardiovascular OR Cardiac Events OR Adverse Cardiac OR Event Coronary Heart Disease OR Coronary Disease OR Angina Pectoris OR atrial fibrillation OR myocardial infarction OR myocardial ischemia OR heart failure OR stroke OR Cerebrovascular Disorders OR peripheral arterial disease OR peripheral vascular disease) 1045782

#2 TS=(telemedicine OR Telemonitoring OR telephone OR eHealth OR Telehealth OR mHealth OR Mobile Health OR Virtual Medicine OR Medicine, Virtual OR smartphone OR smart phone OR Phones, Smart OR Mobile Applications OR Mobile Apps OR Portable Software Apps OR Text Messaging OR text messages OR Texting) 197802

#3 TS=(clinical trial* OR research design OR comparative stud* OR evaluation stud* OR controlled trial* OR follow-up stud* OR prospective stud* OR random* OR placebo* OR single blind* OR double blind*) 4045879

#4 TS=(medication Adherence OR medication Compliance)

#5 #1 AND #2 AND #3 AND #4 964

知网 59

(主题：心血管疾病(精确))OR(主题：动脉粥样硬化性心血管疾病(精确))OR(主题：冠状动脉疾病(精确))OR(主题：卒中(精确))OR(主题：高血压(精确))OR(主题：心房颤动(精确))OR(主题：心力衰竭(精确))OR(主题：心肌梗死(精确))AND((主题：虚拟现实(精确))OR(主题：医疗APP(精确))OR(主题：eHealth(精确))OR(主题：mHealth(精确))OR(主题：移动电话(精确))OR(主题：短信(精确))OR(主题：手机(精确))OR(主题：APP(精确)))AND((主题：随机对照(精确))OR(主题：随机对照(精确))OR(主题：随机(精确))OR(主题：RCT(精确))OR(主题：随机对照(精确))OR(主题：试验(精确))

中国生物医学文献数据库 72

"心血管疾病"[常用字段:智能] OR "动脉粥样硬化性心血管疾病"[常用字段:智能] OR "冠状动脉疾病"[常用字段:智能] OR "卒中"[常用字段:智能] OR "高血压"[常用字段:智能] OR "心房颤动"[常用字段:智能] OR "心力衰竭"[常用字段:智能] OR "心肌梗死"[常用字段:智能] AND "虚拟现实"[常用字段:智能] OR "APP"[常用字段:智能] OR "医疗APP"[常用字段:智能] OR "短信"[常用字段:智能] OR "手机"[常用字段:智能] OR "移动电话"[常用字段:智能] OR "电子邮件"[常用字段:智能] OR "微信"[常用字段:智能] AND "随机"[常用字段:智能] OR "RCT"[常用字段:智能] OR "随机对照"[常用字段:智能] OR "试验"[常用字段:智能]

万方 12

主题:(心血管疾病OR动脉粥样硬化性心血管疾病OR冠状动脉疾病OR卒中OR高血压OR心房颤动OR心力衰竭OR心肌梗死) and 主题:(虚拟现实ORAPPOR医疗APPOR短信OR手机OR移动电话OR电子邮件OR微信) and 主题:(随机ORRCTOR随机对照OR试验)

维普 8

(题名或关键词=心血管疾病OR动脉粥样硬化性心血管疾病OR冠状动脉疾病OR卒中OR高血压OR心房颤动OR心力衰竭OR心肌梗死) AND (题名或关键词=虚拟现实ORAPPOR医疗APPOR短信OR手机OR移动电话OR电子邮件OR微信) AND (任意字段=随机ORRCTOR随机对照OR试验)

**Table S1. PRISMA NMA Checklist of Items to Include When Reporting A Systematic Review Involving a Network Meta-analysis**

| **Section/Topic** | **Item**  **#** | **Checklist Item** | **Reported on Page #** |
| --- | --- | --- | --- |
| **TITLE** | | |  |
| Title | 1 | Identify the report as a systematic review *incorporating a network meta-analysis (or related form of meta-analysis).* |  |
| **ABSTRACT** |  |  | **Page. 1-2** |
| Structured summary | 2 | Provide a structured summary including, as applicable:  **Background:** main objectives  **Methods:** data sources; study eligibility criteria, participants, and interventions; study appraisal; and *synthesis methods, such as network meta-analysis.*  **Results:** number of studies and participants identified; summary estimates with corresponding confidence/credible intervals; *treatment rankings may also be discussed. Authors may choose to summarize pairwise comparisons against a chosen treatment included in their analyses for brevity.*  **Discussion/Conclusions:** limitations; conclusions and implications of findings.  **Other:** primary source of funding; systematic review registration number with registry name. |  |
| **INTRODUCTION** |  |  |  |
| Rationale | 3 | Describe the rationale for the review in the context of what is already known*, including mention of why a network meta- analysis has been conducted.* | **Page. 2-3** |
| Objectives | 4 | Provide an explicit statement of questions being addressed, with reference to participants, interventions, comparisons, outcomes, and study design (PICOS). | **Page. 3** |
| **METHODS** |  |  |  |
| Protocol and registration | 5 | Indicate whether a review protocol exists and if and where it can be accessed (e.g., Web address); and, if available, provide registration information, including registration number. | **Page. 4** |
| Eligibility criteria | 6 | Specify study characteristics (e.g., PICOS, length of follow-up) and report characteristics (e.g., years considered, language, publication status) used as criteria for eligibility, giving rationale. *Clearly describe eligible treatments included in the treatment network, and note whether any have been clustered or merged into the same node (with justification).* | **Page. 5** |
| Information sources | 7 | Describe all information sources (e.g., databases with dates of coverage, contact with study authors to identify additional studies) in the search and date last searched. | **Page. 4** |
| Search | 8 | Present full electronic search strategy for at least one database, including any limits used, such that it could be repeated. | **Page. 4** |
| Study selection | 9 | State the process for selecting studies (i.e., screening, eligibility, included in systematic review, and, if applicable, | **Page. 6** |

| included in the meta-analysis). | | |  |
| --- | --- | --- | --- |
| Data collection process | 10 | Describe method of data extraction from reports (e.g., piloted forms, independently, in duplicate) and any processes for obtaining and confirming data from investigators. | **Page. 6** |
| Data items | 11 | List and define all variables for which data were sought (e.g., PICOS, funding sources) and any assumptions and simplifications made. | **Page. 6** |
| **Geometry of the network** | **S1** | Describe methods used to explore the geometry of the treatment network under study and potential biases related to it. This should include how the evidence base has been graphically summarized for presentation, and what characteristics were compiled and used to describe the evidence base to readers. | **Page. 7** |
| Risk of bias within individual studies | 12 | Describe methods used for assessing risk of bias of individual studies (including specification of whether this was done at the study or outcome level), and how this information is to be used in any data synthesis. | **Page. 7** |
| Summary measures | 13 | State the principal summary measures (e.g., risk ratio, difference in means). *Also describe the use of additional summary measures assessed, such as treatment rankings and surface under the cumulative ranking curve (SUCRA) values, as well as modified approaches used to present summary findings from meta-analyses.* | **Page. 7** |
| Planned methods of analysis | 14 | Describe the methods of handling data and combining results of studies for each network meta-analysis. This should include, but not be limited to:   - *Handling of multi-arm trials;* - *Selection of variance structure;* - *Selection of prior distributions in Bayesian analyses; and* - *Assessment of model fit.* | **Page. 7** |
| **Assessment of Inconsistency** | **S2** | Describe the statistical methods used to evaluate the agreement of direct and indirect evidence in the treatment network(s) studied. Describe efforts taken to address its presence when found. | **Page. 7** |
| Risk of bias across studies | 15 | Specify any assessment of risk of bias that may affect the cumulative evidence (e.g., publication bias, selective reporting within studies). | **Page. 7** |
| Additional analyses | 16 | Describe methods of additional analyses if done, indicating which were pre-specified. This may include, but not be limited to, the following:   - Sensitivity or subgroup analyses; - Meta-regression analyses; - *Alternative formulations of the treatment network; and* - *Use of alternative prior distributions for Bayesian analyses (if applicable).* | **Page. 7** |

| **RESULTS†** | | |  |
| --- | --- | --- | --- |
| Study selection | 17 | Give numbers of studies screened, assessed for eligibility, and included in the review, with reasons for exclusions at each stage, ideally with a flow diagram. | **Page. 8** |
| **Presentation of network structure** | **S3** | Provide a network graph of the included studies to enable visualization of the geometry of the treatment network. | **Figure.2** |
| **Summary of network geometry** | **S4** | Provide a brief overview of characteristics of the treatment network. This may include commentary on the abundance of trials and randomized patients for the different interventions and pairwise comparisons in the network, gaps of evidence in the treatment network, and potential biases reflected by the network structure. | **Page. 12** |
| Study characteristics | 18 | For each study, present characteristics for which data were extracted (e.g., study size, PICOS, follow-up period) and provide the citations. | **Table. 1** |
| Risk of bias within studies | 19 | Present data on risk of bias of each study and, if available, any outcome level assessment. | **Page. 12-13** |
| Results of individual studies | 20 | For all outcomes considered (benefits or harms), present, for each study: 1) simple summary data for each intervention group, and 2) effect estimates and confidence intervals.  *Modified approaches may be needed to deal with information from larger networks.* | **Page. 12-13** |
| Synthesis of results | 21 | Present results of each meta-analysis done, including confidence/credible intervals. *In larger networks, authors may focus on comparisons versus a particular comparator (e.g. placebo or standard care), with full findings presented in an appendix. League tables and forest plots may be considered to summarize pairwise comparisons.* If additional summary measures were explored (such as treatment rankings), these should also be presented. | **Page. 12-13** |
| **Exploration for inconsistency** | **S5** | Describe results from investigations of inconsistency. This may include such information as measures of model fit to compare consistency and inconsistency models, *P* values from statistical tests, or summary of inconsistency estimates from different parts of the treatment network. | **Page. 12-13** |
| Risk of bias across studies | 22 | Present results of any assessment of risk of bias across studies for the evidence base being studied. | **Page. 12-13** |
| Results of additional analyses | 23 | Give results of additional analyses, if done (e.g., sensitivity or subgroup analyses, meta-regression analyses*, alternative network geometries studied, alternative choice of prior distributions for Bayesian analyses,* and so forth). | **Page. 14** |
| **DISCUSSION** |  |  |  |
| Summary of evidence | 24 | Summarize the main findings, including the strength of evidence for each main outcome; consider their relevance to key groups (e.g., healthcare providers, users, and policy- makers). | **Page.16-20** |
| Limitations | 25 | Discuss limitations at study and outcome level (e.g., risk of bias), and at review level (e.g., incomplete retrieval of identified research, reporting bias). *Comment on the validity of the assumptions, such as transitivity and consistency. Comment* | **Page.21** |

| *on any concerns regarding network geometry (e.g., avoidance of certain comparisons).* | | |  |
| --- | --- | --- | --- |
| Conclusions | 26 | Provide a general interpretation of the results in the context of other evidence, and implications for future research. | **Page. 21** |
| **FUNDING** |  |  | **Page. 22** |
| Funding | 27 | Describe sources of funding for the systematic review and other  support (e.g., supply of data); role of funders for the systematic review. This should also include information regarding whether funding has been received from manufacturers of treatments in the network and/or whether some of the authors are content experts with professional conflicts of interest that could affect use of treatments in the network. |  |

PICOS = population, intervention, comparators, outcomes, study design.

* Text in italics indicate S wording specific to reporting of network meta-analyses that has been added to guidance from the PRISMA statement.

† Authors may wish to plan for use of appendices to present all relevant information in full detail for items in this section.

| **Table S2. Description of the medication adherence scales** | | |
| --- | --- | --- |
| Scale | Abbreviation | Brief Description |
| Self-Efficacy for Appropriate Medication Use scale | SEAMS | Risser et al’s SEAMS has good reliability and validity (Cronbach’s alpha=0.89,intraclass correlation  coefficient=0.98) and is composed of 13 items divided into 2 domains[1] Total scores ranged from 13 to 39 points and the scores positively correlated with patients’ self-efficacy for medication adherence, with the higher rating suggesting more medication adherence. |
| Medication Adherence Self-Efficacy scale | MASES | The scale has 26 items rated on a 3-point Likert-type scale; a total mean score is calculated by averaging responses on all items. Higher scores indicate a greater level of medication adherence self-efficacy. The scale has 26 items rated on a 3-point Likert-type scale; a total mean score is calculated by averaging responses on all items. Higher scores indicate a greater level of medication adherence self-efficacy. Higher scores indicate a greater level of medication adherence[2]. The Cronbach’s alpha for the entire 26-item scale was .95. |
| Morisky Medication Adherence Scale-8 | MMAS-8 | Medication adherence was measured by using a validated 8-item Morisky Medication Adherence Scale (MMAS-8). The 8 items of MMAS-8 were designed to detect patients who are nonadherent  to their medications and the reasons for such behavior, such as forgetfulness, insufficient knowledge, inconvenience, and side effects. The score of MMAS-8 ranges from 0 to 8, with a score of <6 indicating low adherence, 6 to <8 indicating medium Adherence, and 8 indicating high adherence[3]. |
| Morisky Medication Adherence Scale-4 | MMAS-4 | The Morisky Medication Adherence Scale-4 (MMAS-4) is a shortened version of the original Morisky Medication Adherence Scale (MMAS). The MMAS-4 consists of four simple yes-or-no questions that help healthcare providers or researchers gauge a patient's medication adherence behavior. Scores range from 0 to 4, with higher scores indicating greater medication adherence. |
| Health Promoting Lifestyle Profile II | HPLP II | Medication adherence were assessed using the Chinese version of the Health Promoting Lifestyle Profile II (HPLP II), which have a 4-point ordinal response format. Higher scores indicate better health behaviors[4]. |
| Morisky Medication Adherence Scale-7 | MMAS-7 | Medication adherence was assessed using a 7-item version of the 8-item Morisky Medication Adherence Scale. Scores could range from 0 to 7, with higher scores indicating better adherence. |
| Medication Adherence Report  Scale-5 questionnaire | MARS-5 | The MARS-5 assesses a patient’s typical medication adherence through 5 questions. Responses are summed for a total score ranging between 5 and 25, with higher scores indicating a higher level of adherence[5]. |

References

[1]. Risser J, Jacobson TA, Kripalani S: **Development and psychometric evaluation of the Self-efficacy for Appropriate Medication Use Scale (SEAMS) in low-literacy patients with chronic disease**. *Journal of nursing measurement* 2007, **15**(3):203-219.

[2]. Ogedegbe G, Mancuso CA, Allegrante JP, Charlson ME: **Development and evaluation of a medication adherence self-efficacy scale in hypertensive African-American patients**. *Journal of clinical epidemiology* 2003, **56**(6):520-529.

[3]. Morisky DE, Ang A, Krousel-Wood M, Ward HJ: **Predictive validity of a medication adherence measure in an outpatient setting**. *Journal of clinical hypertension (Greenwich, Conn)* 2008, **10**(5):348-354.

[4]. Kuster AE, Fong CM: **Further psychometric evaluation of the Spanish language health-promoting lifestyle profile**. *Nursing research* 1993, **42**(5):266-269.

[5]. Stone JK, Shafer LA, Graff LA, Lix L, Witges K, Targownik LE, Haviva C, Sexton K, Bernstein CN: **Utility of the MARS-5 in Assessing Medication Adherence in IBD**. *Inflamm Bowel Dis* 2021, **27**(3):317-324.

| **Table S3. Quality of evidence with GRADE assessment for medication adherence.** | | | | | | | |
| --- | --- | --- | --- | --- | --- | --- | --- |
| Comparison | Network SMD^a^  (95%CI^b^) | Risk of  bias | Inconsistency | Indirectness | Imprecision | Publication  bias | Overall |
| Combined intervention vs Usual care | 0.89  (0.22, 1.57) | Serious | Not serious | Not serious | Not serious | Strongly  suspected | Low |
| Telemonitoring vs Usual care | 0.70 (0.02,1.39) | Serious | Not serious | Not serious | Not serious | Strongly  suspected | Low |
| Telephone support vs Usual care | 0.68 (0.02,1.33) | Serious | Not serious | Not serious | Not Serious | Strongly  suspected | Low |
| APP intervention vs Usual care | 0.65  (0.01, 1.30) | Serious | Not serious | Not serious | Not serious | Strongly  suspected | Low |
| SMS intervention vs Usual care | 0.28 (-0.20,0.77) | Serious | Not serious | Not serious | Serious | Strongly  suspected | Very low |
| Combined intervention vs SMS intervention | 0.61  (-0.22, 1.44) | Serious | Not serious | Not serious | Serious | Strongly  suspected | Very low |
| Telemonitoring vs SMS intervention | 0.42  (-0.34, 1.18) | Serious | Not serious | Not serious | Serious | Strongly  suspected | Very Low |
| Telephone support vs SMS intervention | 0.39 (-0.42,1.21) | Serious | Not serious | Not serious | Serious | Strongly  suspected | Very Low |
| APP intervention vs SMS intervention | 0.37  (-0.44, 1.18) | Serious | Not serious | Not serious | Serious | Strongly  suspected | Very Low |
| Combined intervention vs APP intervention | 0.24  (-0.69, 1.17) | Serious | Not serious | Not serious | Serious | Strongly  suspected | Very low |
| Telemonitoring vs APP intervention | 0.05  (-0.89, 0.99) | Serious | Not serious | Not serious | Serious | Strongly  suspected | Very low |
| Telephone support vs APP intervention | 0.02  (-0.90, 0.94) | Not serious | Not serious | Not serious | Serious | Strongly  suspected | Low |
| Combined intervention vs Telephone support | 0.22  (-0.59, 1.02) | Serious | Not serious | Not serious | Serious | Strongly  suspected | Very low |
| Telemonitoring vs Telephone support | 0.03  (-0.92, 0.97) | Serious | Not serious | Not serious | Serious | Strongly  suspected | Very low |
| Combined intervention vs Telemonitoring | 0.19  (-0.77, 1.15) | Not serious | Not serious | Not serious | Serious | Strongly  suspected | Low |
| ^a^SMD: standardized mean difference.  ^b^CI: confidence interval. | | | | | | | |

| **Table S4. Quality of evidence with GRADE SBP.** | | | | | | | |
| --- | --- | --- | --- | --- | --- | --- | --- |
| Comparison | Network SMD^a^  (95%CI^b^) | Risk of  bias | Inconsistency | Indirectness | Imprecision | Publication  bias | Overall |
| Combined intervention vs Usual care | -1.21  (-2.12,-0.31) | Serious | Not serious | Not serious | Not serious | Not serious | Moderate |
| Telemonitoring vs Usual care | -0.21 (-1.13,0.71) | Serious | Not serious | Not serious | Serious | Not serious | Low |
| Telephone support vs Usual care | -0.75 (-2.23,0.73) | Serious | Not serious | Not serious | Serious | Not serious | Low |
| APP intervention vs Usual care | -0.74 (-1.57,0.09) | Serious | Not serious | Not serious | Serious | Not serious | Low |
| SMS intervention vs Usual care | -0.14 (-0.72,0.45) | Serious | Not serious | Not serious | Serious | Not serious | Low |
| Combined intervention vs SMS intervention | -1.08 (-2.15,-0.01) | Serious | Not serious | Not serious | Not serious | Not serious | Moderate |
| Telemonitoring vs SMS intervention | -0.07 (-1.16,1.01) | Serious | Not serious | Not serious | Serious | Not serious | Low |
| Telephone support vs SMS intervention | -0.61 (-2.21,0.98) | Serious | Not serious | Not serious | Serious | Not serious | Low |
| APP intervention vs SMS intervention | -0.60 (-1.61,0.41) | Serious | Not serious | Not serious | Serious | Not serious | Low |
| Combined intervention vs Telemonitoring | -1.00 (-2.29,0.28) | Serious | Not serious | Not serious | Serious | Not serious | Low |
| APP intervention vs Telemonitoring | -0.53 (-1.76,0.70) | Serious | Not serious | Not serious | Serious | Not serious | Low |
| Telephone support vs Telemonitoring | -0.54 (-2.28,1.21) | Serious | Not serious | Not serious | Serious | Not serious | Low |
| Combined intervention vs Telephone support | -0.46 (-1.64,0.71) | Not serious | Not serious | Not serious | Serious | Not serious | Moderate |
| APP intervention vs Telephone support | 0.01 (-1.69,1.71) | Not serious | Not serious | Not serious | Serious | Not serious | Moderate |
| Combined intervention vs APP intervention | -0.48 (-1.70,0.75) | Not serious | Not serious | Not serious | Serious | Not serious | Moderate |
| ^a^SMD: standardized mean difference.  ^b^CI: confidence interval. | | | | | | | |

| **Table S5. Quality of evidence with GRADE assessment for DBP.** | | | | | | | |
| --- | --- | --- | --- | --- | --- | --- | --- |
| Comparison | Network SMD^a^  (95%CI^b^) | Risk of  bias | Inconsistency | Indirectness | Imprecision | Publication  bias | Overall |
| Usual care vs Combined intervention | -0.95 (-1.64,-0.27) | Serious | Not serious | Not serious | Not serious | Not serious | Moderate |
| SMS intervention vs Combined intervention | -0.90 (-1.70,-0.10) | Serious | Not serious | Not serious | Not serious | Not serious | Moderate |
| Telemonitoring vs Combined intervention | -0.73 (-1.82,0.36) | Serious | Not serious | Not serious | Serious | Not serious | Low |
| Telephone support vs Combined intervention | -0.32 (-1.17,0.52) | Serious | Not serious | Not serious | Serious | Not serious | Low |
| APP intervention vs Combined intervention | -1.13 (-2.11,-0.16) | Serious | Not serious | Not serious | Not serious | Not serious | Moderate |
| Usual care vs APP intervention | -0.18 (-0.87,0.51) | Serious | Not serious | Not serious | Serious | Not serious | Low |
| SMS intervention vs APP intervention | -0.24 (-1.04,0.57) | Serious | Not serious | Not serious | Serious | Not serious | Low |
| Telemonitoring vs APP intervention | -0.41 (-1.50,0.69) | Serious | Not serious | Not serious | Serious | Not serious | Low |
| Telephone support vs App intervention | -0.81 (-2.10,0.48) | Serious | Not serious | Not serious | Serious | Not serious | Low |
| Usual care vs Telephone support | -0.63 (-1.72,0.46) | Serious | Not serious | Not serious | Serious | Not serious | Low |
| SMS intervention vs Telephone support | -0.57 (-1.74,0.59) | Serious | Not serious | Not serious | Serious | Not serious | Low |
| Telemonitoring vs Telephone support | -0.40 (-1.78,0.97) | Serious | Not serious | Not serious | Serious | Not serious | Low |
| Usual care vs Telemonitoring | -0.22 (-1.07,0.62) | Not serious | Not serious | Not serious | Serious | Not serious | Moderate |
| SMS intervention vs Telemonitoring | -0.17 (-1.11,0.77) | Not serious | Not serious | Not serious | Serious | Not serious | Moderate |
| Usual care vs SMS intervention | -0.05 (-0.47,0.36) | Not serious | Not serious | Not serious | Serious | Not serious | Moderate |
| ^a^SMD: standardized mean difference.  ^b^CI: confidence interval. | | | | | | | |

**Table S6. Relative effect sizes of SBP^a^ and DBP^b^ at post-intervention according to network meta-analysis.**

| **Combined intervention** | **-1.13 (-2.11,-0.16)** | -0.32 (-1.17,0.52) | -0.73 (-1.82,0.36) | **-0.90 (-1.70,-0.10)** | **-0.95 (-1.64,-0.27)** |
| --- | --- | --- | --- | --- | --- |
| -0.48 (-1.70,0.75) | **APP^c^** | -0.81 (-2.10,0.48) | -0.41 (-1.50,0.69) | -0.24 (-1.04,0.57) | -0.18 (-0.87,0.51) |
| -0.46 (-1.64,0.71) | 0.01 (-1.69,1.71) | **Telephone support** | -0.40 (-1.78,0.97) | -0.57 (-1.74,0.59) | -0.63 (-1.72,0.46) |
| -1.00 (-2.29,0.28) | -0.53 (-1.76,0.70) | -0.54 (-2.28,1.21) | **Telemonitoring** | -0.17 (-1.11,0.77) | -0.22 (-1.07,0.62) |
| **-1.08 (-2.15,-0.01)** | -0.60 (-1.61,0.41) | -0.61 (-2.21,0.98) | -0.07 (-1.16,1.01) | **SMS^d^** | -0.05 (-0.47,0.36) |
| **-1.21**  **(-2.12,-0.31)** | -0.74 (-1.57,0.09) | -0.75 (-2.23,0.73) | -0.21 (-1.13,0.71) | -0.14 (-0.72,0.45) | **Usual care** |

The data on the lower left is SBP, and the data on the upper right is DBP. Bold indicates statistically significant differences between the two groups.

^a^SBP: Systolic blood pressure.

^b^DBP: Diastolic blood pressure.

^c^APP: Mobile phone applications.

^d^SMS, Short messaging service.

**Table S7. Relative effect sizes of subgroups according to network meta-analysis.**

**Table S7(A). Relative effect sizes of duration of intervention according to network meta-analysis.**

| **Telephone support** | - | - | - | - | - |
| --- | --- | --- | --- | --- | --- |
| 0.01 (-0.76,0.78) | **APP^a^** | 0.40 (-0.57,1.37) | 1.55 (0.58,2.52) | 0.66 (-0.05,1.37) | **1.05 (0.42,1.67)** |
| 0.12 (-0.70,0.94) | 0.11 (-0.75,0.97) | **Telemonitori-ng** | 1.15 (0.11,2.20) | 1.06 (0.25,1.87) | **1.45 (0.71,2.18)** |
| 0.17 (-0.49,0.83) | 0.16 (-0.67,1.00) | 0.05 (-0.83,0.93) | **Combined intervention** | 2.21 (1.40,3.03) | **2.60 (1.85,3.34)** |
| 0.35 (-0.36,1.06) | 0.34 (-0.42,1.10) | 0.23 (-0.47,0.92) | 0.18 (-0.60,0.95) | **SMS^b^** | **0.38 (0.05,0.72)** |
| **0.52 (0.01,1.03)** | 0.51 (-0.07,1.09) | 0.40 (-0.24,1.04) | 0.35 (-0.25,0.95) | 0.17 (-0.32,0.66) | **Usual care** |

The data on the lower left are duration < 3 months interventions, and the data on the upper right are duration ≥ 3 months interventions. Bold indicates statistically significant differences between the two groups.

^a^APP: Mobile phone applications.

^b^SMS: Short messaging service.

**Table S7(B). Relative effect sizes of gender according to network meta-analysis.**

| **APP^a^** | 0.36 (-1.32,2.04) | 0.11 (-1.64,1.86) | **2.27 (0.17,4.36)** | 0.47 (-1.28,2.22) | 0.33 (-1.09,1.75) |
| --- | --- | --- | --- | --- | --- |
| -0.03 (-1.04,0.99) | **Telemonitori-ng** | 0.47 (-0.88,1.82) | 0.36 (-1.32,2.04) | 0.11 (-1.25,1.47) | 0.69 (-0.20,1.58) |
| 0.44 (-0.15,1.03) | 0.46 (-0.36,1.29) | **SMS^b^** | 2.38 (0.53,4.22) | 0.58 (-0.86,2.02) | 0.22 (-0.79,1.24) |
| 0.52 (-0.16,1.20) | 0.54 (-0.48,1.57) | 0.08 (-0.53,0.69) | **Combined intervention** | 1.80 (-0.05,3.64) | **2.60 (1.06,4.13)** |
| 0.57 (-0.19,1.33) | 0.60 (-0.48,1.67) | 0.13 (-0.56,0.83) | 0.05 (-0.52,0.63) | **Telephone support** | 0.80 (-0.22,1.83) |
| **0.76 (0.29,1.23)** | 0.79 (-0.11,1.69) | 0.32 (-0.03,0.68) | 0.25 (-0.24,0.73) | 0.19 (-0.40,0.79) | **Usual care** |

The data on the lower left are male to female ratio ≥ 1 studies, and the data on the upper right are male to female ratio < 1 studies. Bold indicates statistically significant differences between the two groups.

^a^APP: Mobile phone applications.

^b^SMS, Short messaging service.

**Table S7(C). Relative effect sizes of based on theory interventions according to network meta-analysis.**

| **Telemonitori-ng** | 0.07 (-1.13,1.27) | 0.19 (-0.78,1.16) | 0.80 (-0.53,2.14) | 0.45 (-0.43,1.33) | 0.68 (-0.66,2.01) |
| --- | --- | --- | --- | --- | --- |
| 0.40 (-0.61,1.41) | **APP^a^** | 0.26 (-0.83,1.36) | 0.13 (-1.29,1.55) | 0.26 (-0.83,1.36) | 0.60 (-0.69,1.89) |
| **1.23 (0.40,2.05)** | **0.83 (0.10,1.56)** | **SMS^b^** | 0.99 (-0.25,2.24) | 0.26 (-0.46,0.99) | 0.87 (-0.37,2.10) |
| **1.24 (0.29,2.19)** | 0.84 (-0.03,1.71) | 0.01 (-0.64,0.67) | **Combined intervention** | **1.26 (0.25,2.26)** | 0.13 (-1.29,1.55) |
| **1.45 (0.68,2.21)** | **1.05 (0.39,1.71)** | 0.22 (-0.09,0.53) | 0.20 (-0.36,0.77) | **Usual care** | **1.13 (0.13,2.13)** |
| **1.51 (0.62,2.41)** | **1.11 (0.31,1.92)** | 0.29 (-0.28,0.85) | 0.27 (-0.22,0.76) | 0.07 (-0.40,0.53) | **Telephone support** |

The data on the lower left are based on theory interventions, and the data on the upper right are not based on theory interventions. Bold indicates statistically significant differences between the two groups.

^a^APP: Mobile phone applications.

^b^SMS, Short messaging service.


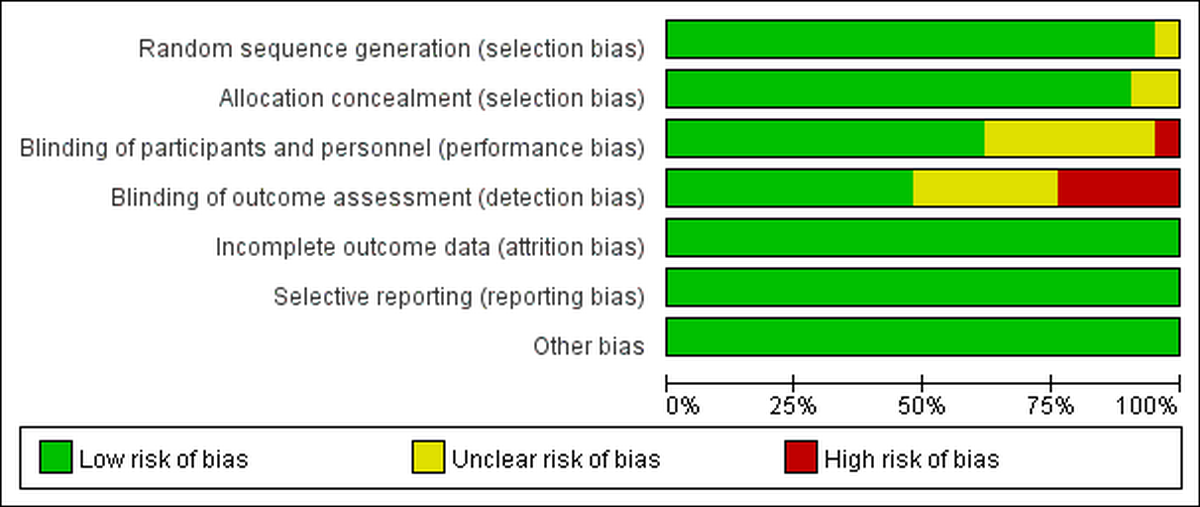


**Figure S1(A).** Risk of bias graph about each risk of bias item presented as percentages across all included studies.


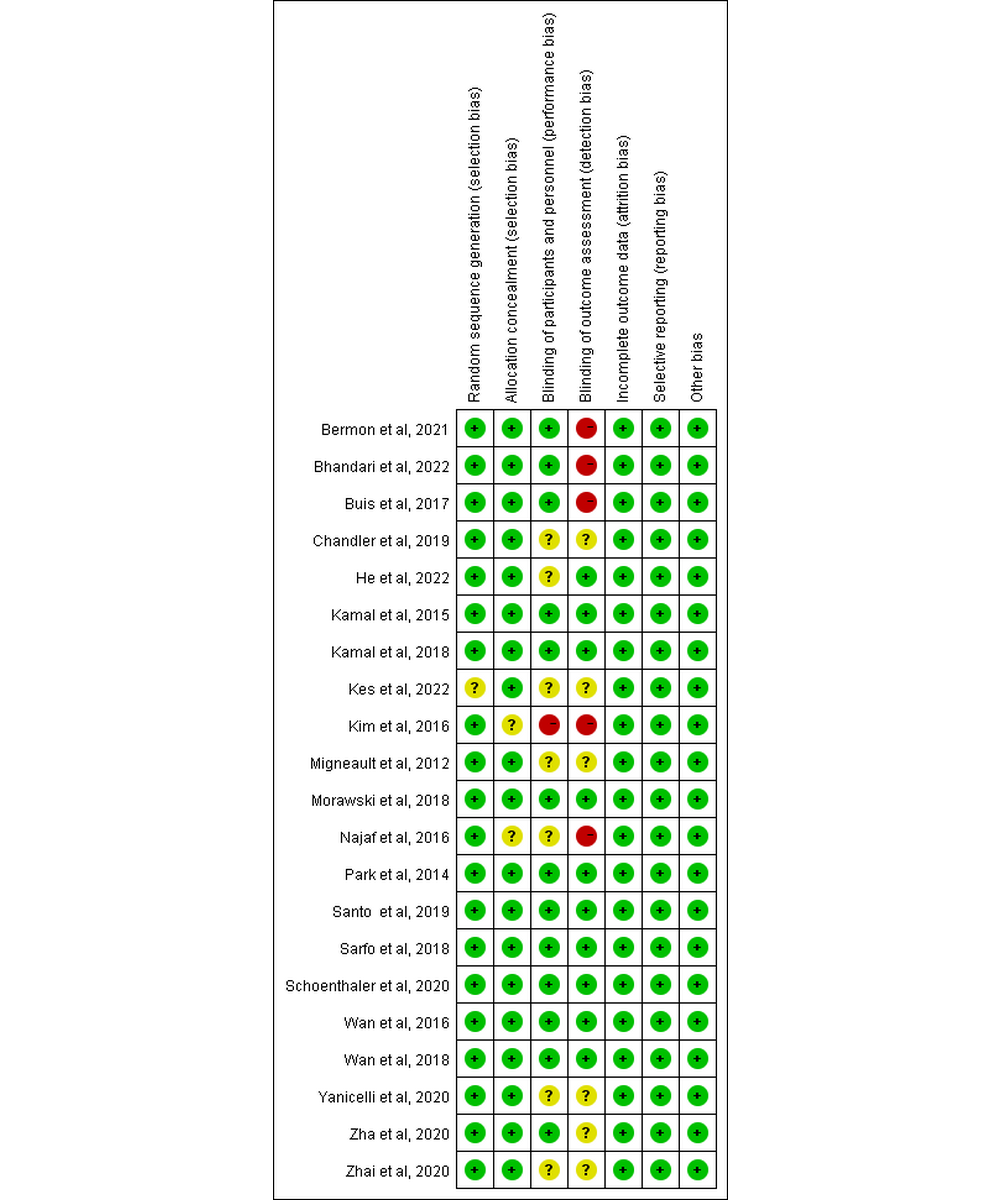


**Figure S1(B).** Summary of risk of bias for each trial.


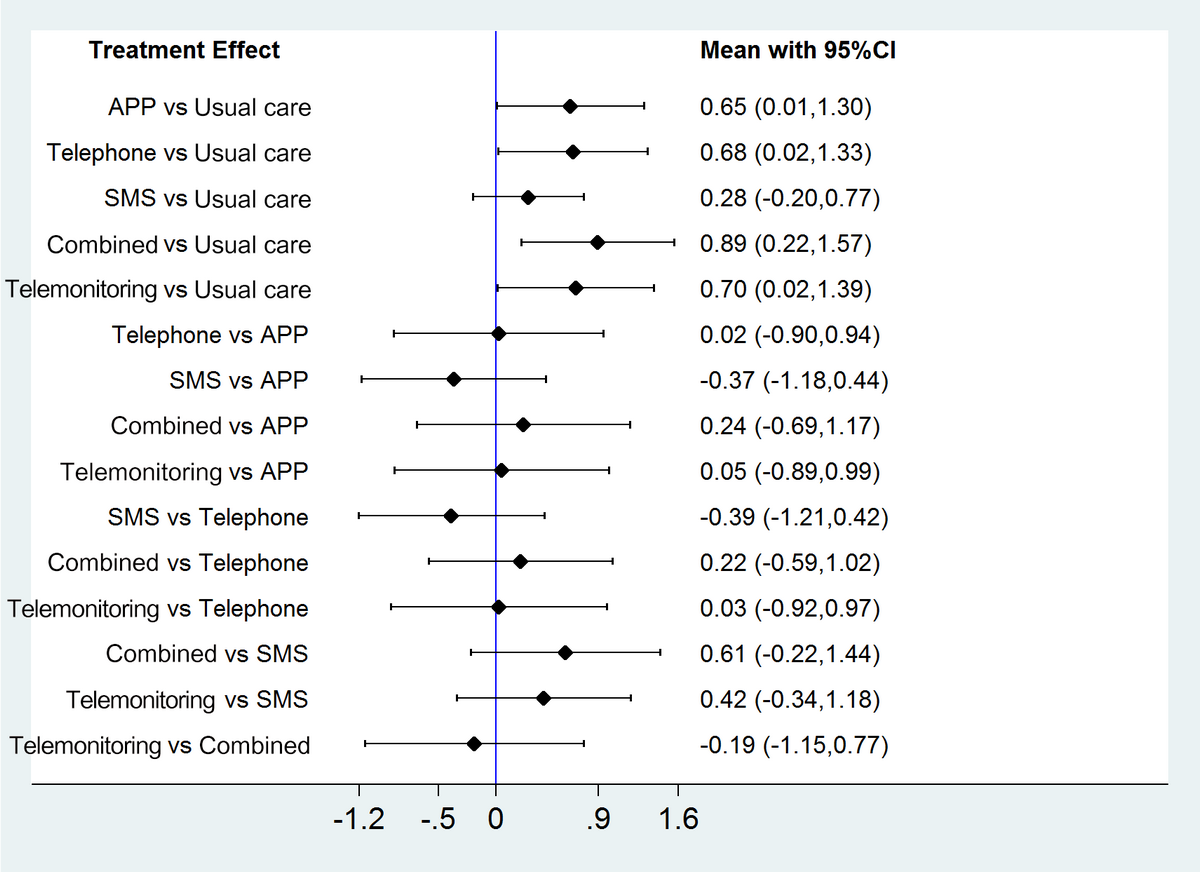


**Figure S2.** The forest plot for pairwise comparison of medication adherence. APP, mobile phone applications; SMS, short messaging service.


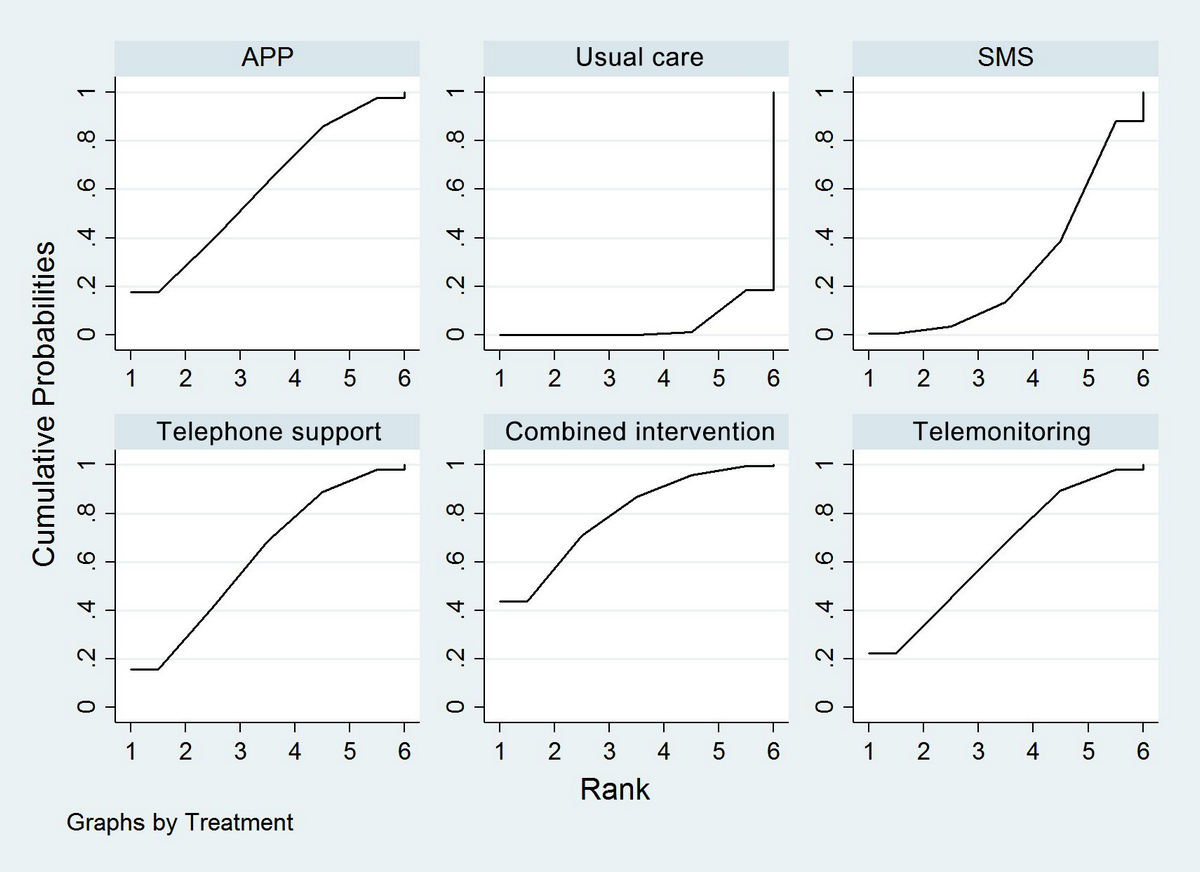


**Figure S3.** The surface under cumulative ranking curve of medication adherence. APP, mobile phone applications; SMS, short messaging service.


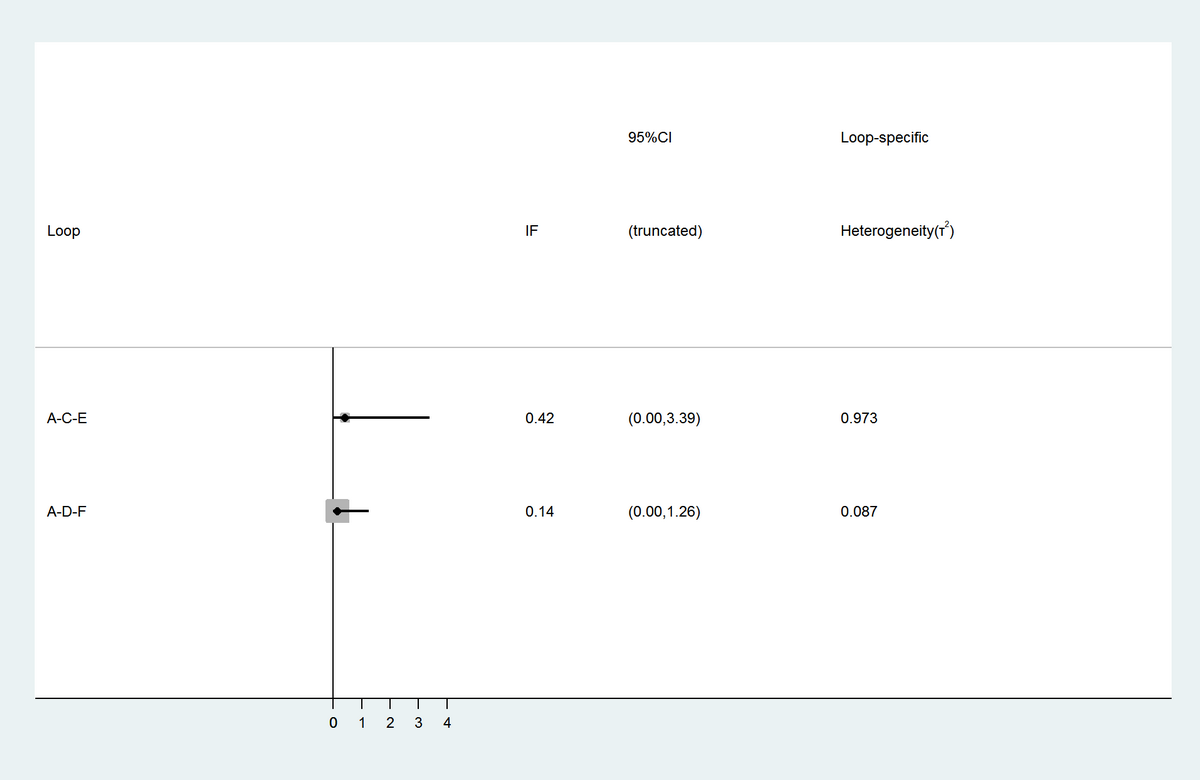


**Figure S4.** The loop inconsistency plot.


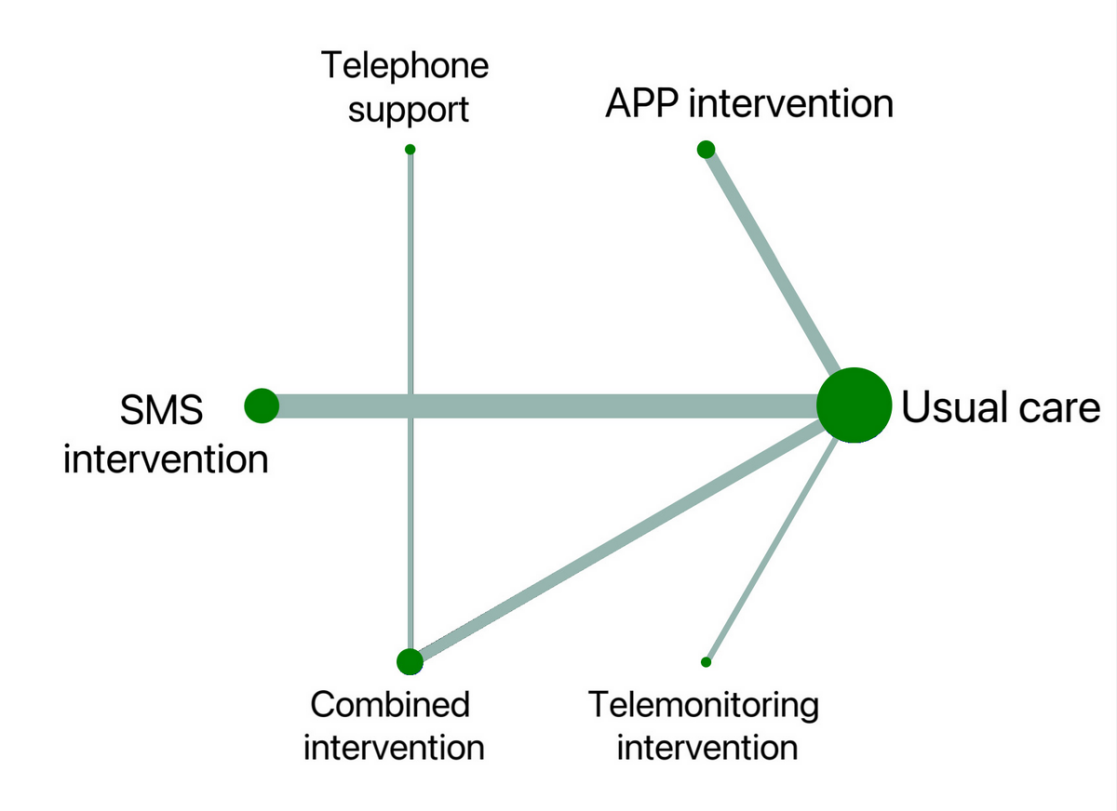


**Figure S5(A).** The network plots regarding systolic blood pressure. APP, mobile phone applications; SMS, short messaging service.


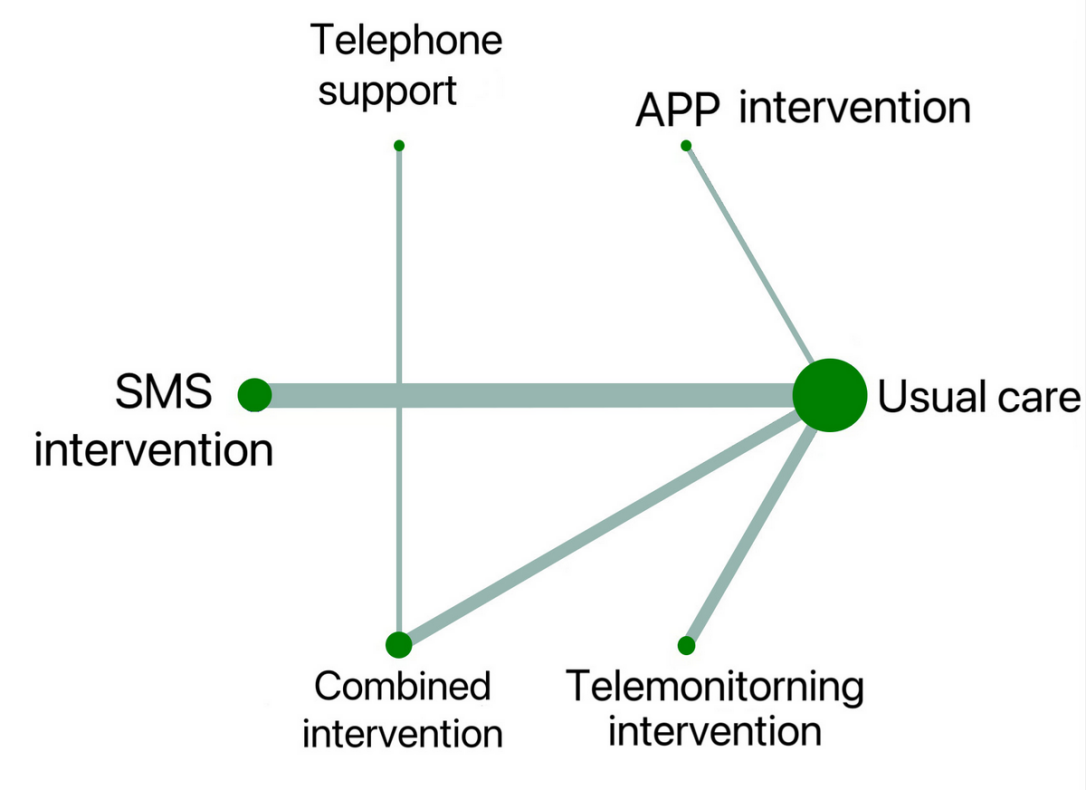


**Figure S5(B)**. The network plots regarding diastolic blood pressure. APP, mobile phone applications; SMS, short messaging service.


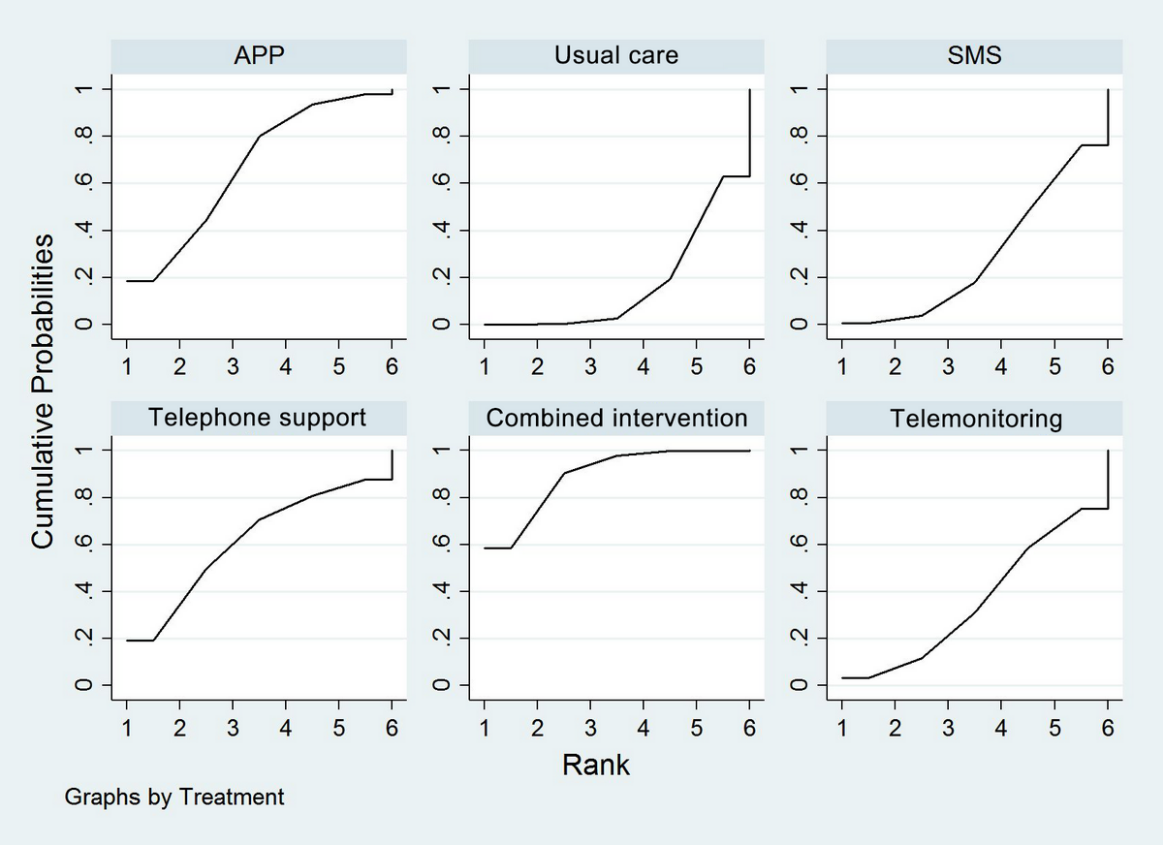


**Figure S6 (A).** The surface under cumulative ranking curve of systolic blood pressure. APP, mobile phone applications; SMS, short messaging service.


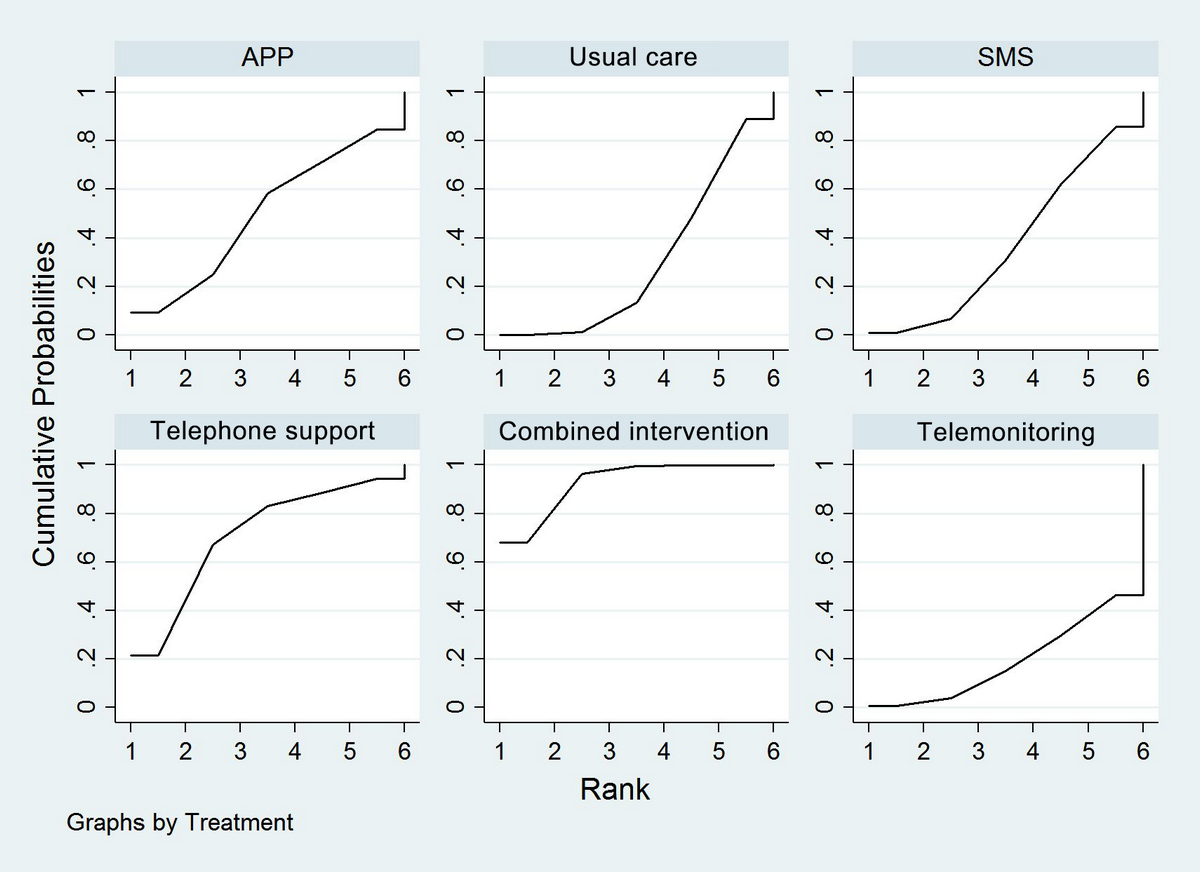


**Figure S6 (B).** The surface under cumulative ranking curve of diastolic blood pressure. APP, mobile phone applications; SMS, short messaging service.


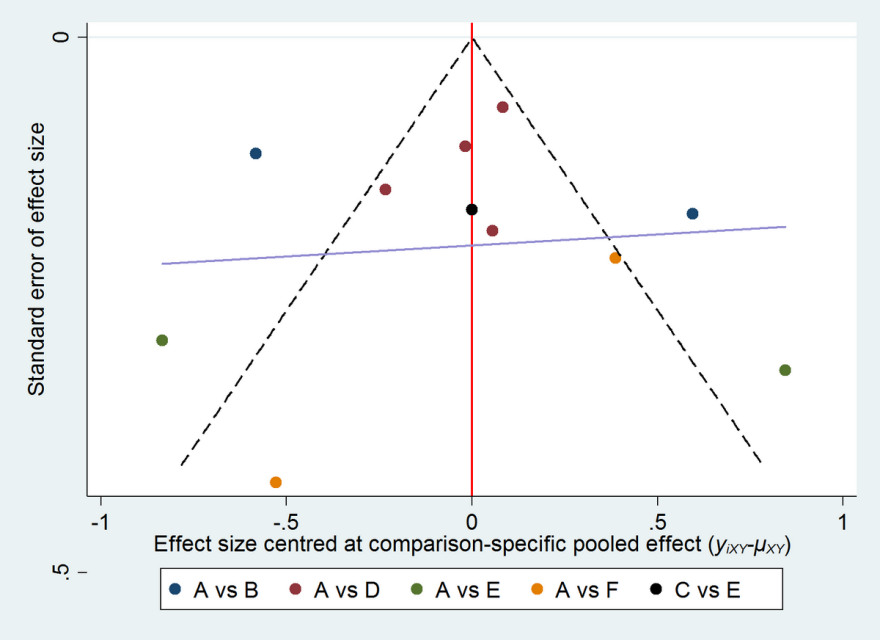


**Figure S7(A).** Funnel plots of systolic blood pressure.


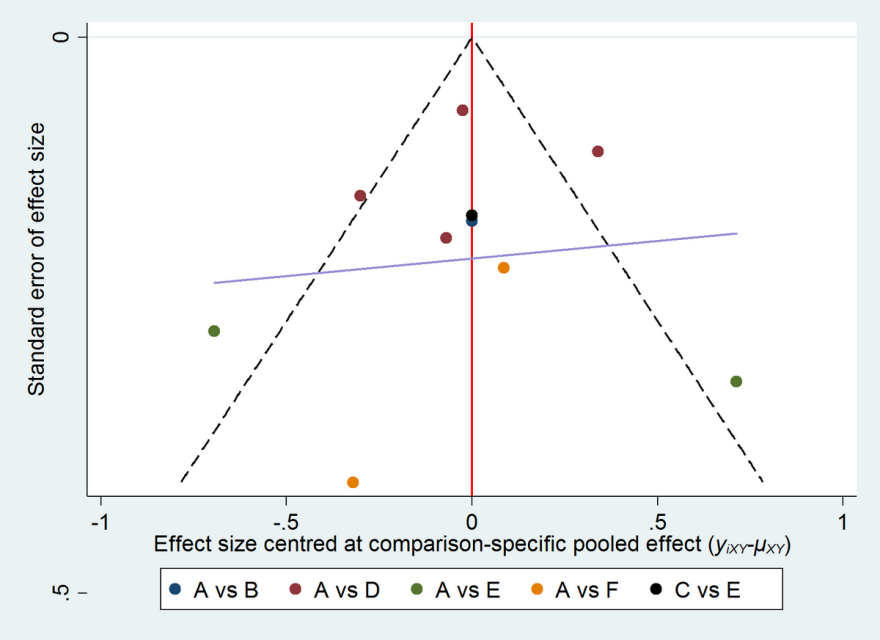


**Figure S 7(B).** Funnel plots of diastolic blood pressure.
